# Genome-wide multi-omics profiling of the 8p11-p12 amplicon in breast carcinoma

**DOI:** 10.18632/oncotarget.25329

**Published:** 2018-05-08

**Authors:** Toshima Z. Parris, Elisabeth Werner Rönnerman, Hanna Engqvist, Jana Biermann, Katarina Truvé, Szilárd Nemes, Eva Forssell-Aronsson, Giovanni Solinas, Anikó Kovács, Per Karlsson, Khalil Helou

**Affiliations:** ^1^ Department of Oncology, Institute of Clinical Sciences, Sahlgrenska Cancer Center, Sahlgrenska Academy at University of Gothenburg, Gothenburg, Sweden; ^2^ Sahlgrenska University Hospital, Department of Clinical Pathology and Genetics, Gothenburg, Sweden; ^3^ Bioinformatics Core Facility, Sahlgrenska Academy at University of Gothenburg, Gothenburg, Sweden; ^4^ Swedish Hip Arthroplasty Register, Gothenburg, Sweden; ^5^ Department of Radiation Physics, Institute of Clinical Sciences, Sahlgrenska Cancer Center, Sahlgrenska Academy at University of Gothenburg, Gothenburg, Sweden; ^6^ The Wallenberg Laboratory, Department of Molecular and Clinical Medicine, University of Gothenburg, Gothenburg, Sweden

**Keywords:** breast cancer, genomic instability, 8p11-p12 amplification, genomic profiling, molecular subtype

## Abstract

Genomic instability contributes to the neoplastic phenotype by deregulating key cancer-related genes, which in turn can have a detrimental effect on patient outcome. DNA amplification of the 8p11-p12 genomic region has clinical and biological implications in multiple malignancies, including breast carcinoma where the amplicon has been associated with tumor progression and poor prognosis. However, oncogenes driving increased cancer-related death and recurrent genetic features associated with the 8p11-p12 amplicon remain to be identified. In this study, DNA copy number and transcriptome profiling data for 229 primary invasive breast carcinomas (corresponding to 185 patients) were evaluated in conjunction with clinicopathological features to identify putative oncogenes in 8p11-p12 amplified samples. Illumina paired-end whole transcriptome sequencing and whole-genome SNP genotyping were subsequently performed on 23 samples showing high-level regional 8p11-p12 amplification to characterize recurrent genetic variants (SNPs and indels), expressed gene fusions, gene expression profiles and allelic imbalances. We now show previously undescribed chromothripsis-like patterns spanning the 8p11-p12 genomic region and allele-specific DNA amplification events. In addition, recurrent amplification-specific genetic features were identified, including genetic variants in the *HIST1H1E* and *UQCRHL* genes and fusion transcripts containing *MALAT1* non-coding RNA, which is known to be a prognostic indicator for breast cancer and stimulated by estrogen. In summary, these findings highlight novel candidate targets for improved treatment of 8p11-p12 amplified breast carcinomas.

## INTRODUCTION

Molecular profiling of cancer genomes and epigenomes with microarray and next-generation sequencing (NGS) technologies has, in recent years, provided a more in-depth overview of disease-specific aberrations, thereby identifying novel targets for treatment. These complex landscapes of somatic structural rearrangements and epigenomic modulations are comprised of a composite of driver and bystander aberrations either acquired via chromothripsis or accumulated over time [1, 2]. Nevertheless, certain structural variants (SVs) confer selective advantage because they contain one or more genes with tumorigenic potential [3]. One such recurrent genetic aberration is DNA amplification of the 8p11-p12 genomic region, which has clinical and biological implications in multiple malignancies [4, 5]. In breast carcinoma (both familial and sporadic cases), the 8p11-p12 genomic region is a frequent target for DNA amplification and loss, resulting in the deregulation of multiple putative “driver” genes and aggressive tumor features [6–10]. However, the 8p11-p12 genomic region spans over 10 Megabases (Mb) and encompasses over 50 known genes, many of which have been shown to be activated by more than one molecular mechanism, *i.e.* translocation and DNA amplification [11]. Therefore, the aggressive phenotype imposed by the 8p11-p12 amplicon may be the result of one or more interacting genes in this genomic region and/or crosstalk with other genetic and epigenomic aberrations [12]. However, little is known about the type and extent of other structural rearrangements (translocations and fusion genes) and genetic variants (indels and substitutions) found in 8p11-p12 amplified tumors and their contribution to aggressive features.

In this study, we evaluated array-CGH and gene expression microarray data for 229 breast cancer patients in relation to clinicopathological features and clinical outcome to identify putative oncogenes and tumor suppressors associated with 8p11-p12 amplification [12]. Furthermore, we performed RNA sequencing (RNA-seq) in conjunction with SNP genotyping analysis for 23 amplified tumors to identify common chromosomal rearrangements and genetic variants.

## RESULTS

### DNA profiling reveals chromothripsis-like events spanning regions of amplification in breast carcinoma

**Figure 1:**
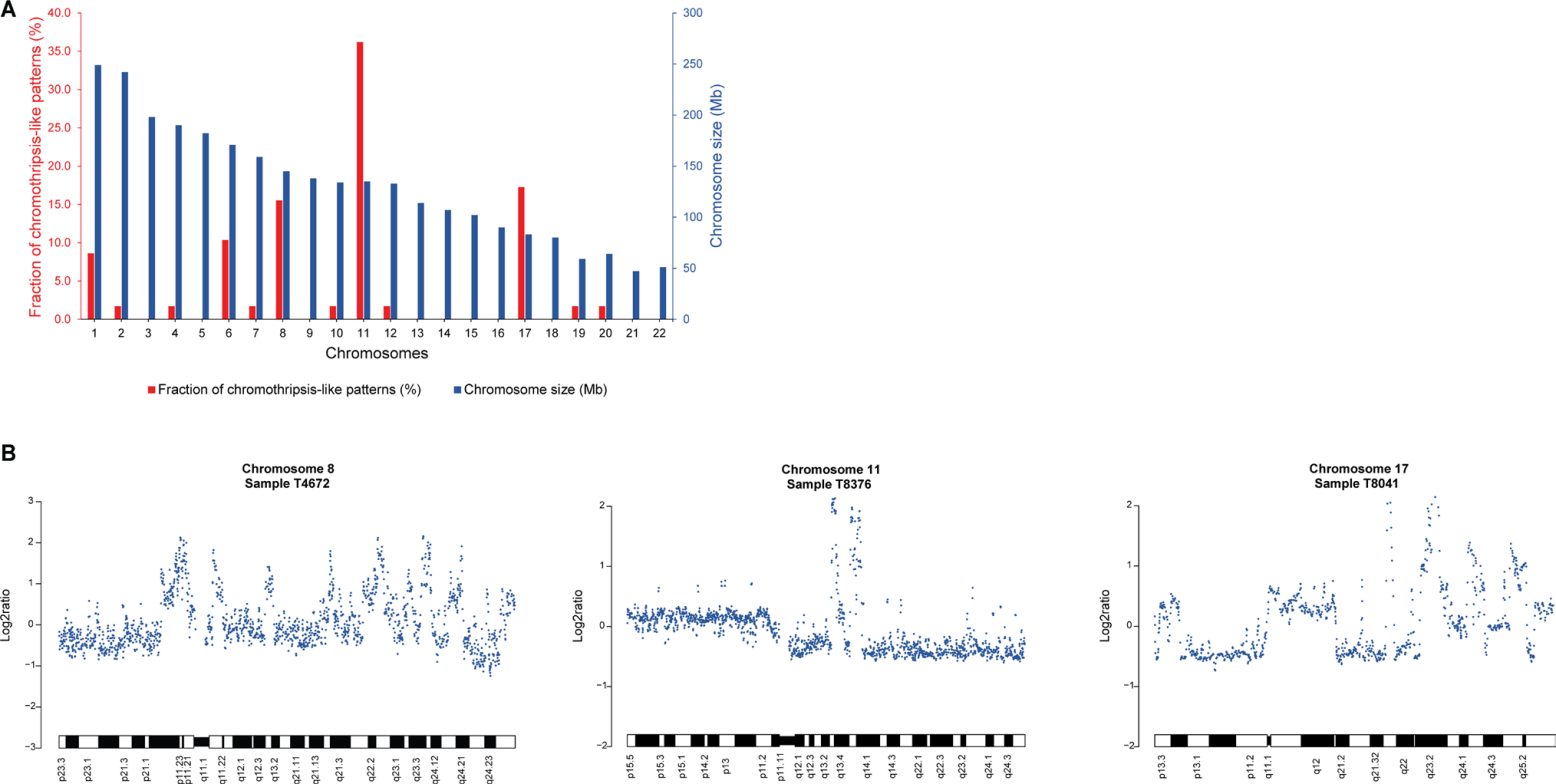
Chromothripsis-like events frequently occur at chromosomes 11, 17, and 8 in breast carcinoma (**A**) Frequency of CTLP regions in the genome. Red and blue bars indicate the fraction of chromothripsis-like regions in percent and chromosome size in megabases, respectively. (**B**) Three representative examples of chromothripsis-like patterns in array-CGH DNA copy number profiles from the 229 breast carcinoma samples. The x-axis depicts the genomic location and the log_2_ratio on the y-axis.

In a genome-wide screen for copy number alterations (CNAs), array comparative genomic hybridization (array-CGH) data for 229 invasive breast carcinomas were analyzed using the Rank Segmentation algorithm in Nexus Copy Number. Recurrent genomic regions of high-level DNA amplification were observed with a frequency of ≥ 10% (*P* < 0.01) at 1q, 8p12-p11.21, 8q11.21-q11.23, 8q11.23-q24.3, 11q13.3-q13.4, and 17q23.3. Chromothripsis-like patterns (CTLP) for genomic gains and losses were then identified in the dataset with ≥ 20 changes in estimated copy number state. In total, 58 CTLPs were observed in 49 of the 229 samples (21%), with 9/49 samples (18%) involving chromothripsis-like events on two different chromosomes (Supplementary Table 1). On average, CTLPs involved 33 changes in copy number state (range, 20–95 changes) and spanned 86.7 Mb (range, 30–243 Mb), affecting localized genomic regions, chromosome arms and whole chromosomes. In agreement with a study on CTLP in breast carcinomas [13], chromothripsis-like events were observed primarily on chromosomes 1, 6, 8, 11, and 17 (Figure 1). In the present study, CTLPs were most prevalent on chromosomes 11 (36%), 17 (17%), and 8 (16%), spanning several of the detected genomic regions of DNA amplification on 11q, 17q, 8p, and 8q.

### Nine minimal common 8p11-p12 amplification peaks identified using DNA copy number analysis

**Figure 2:**
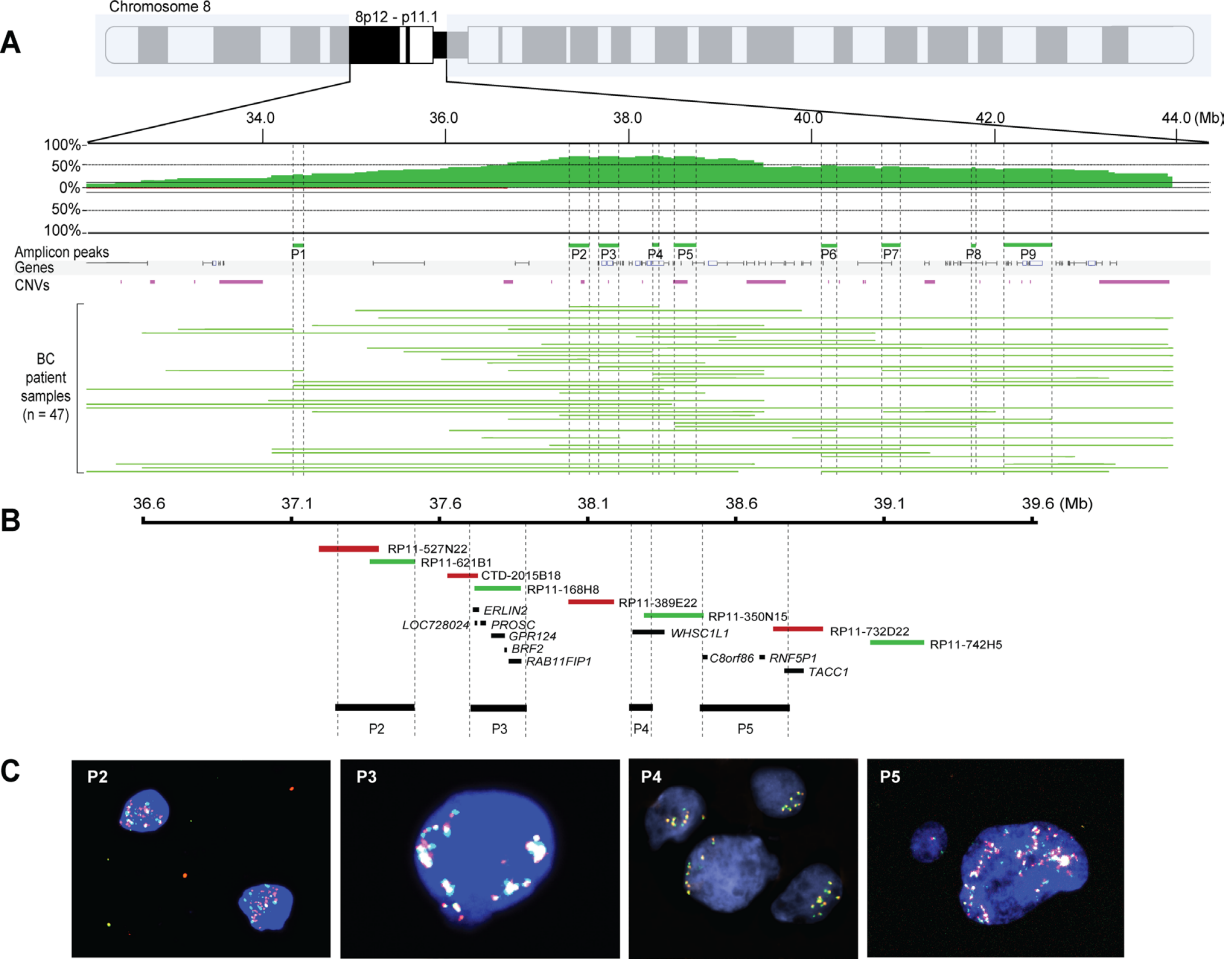
Nine common 8p11-p12 amplification peaks identified despite breast tumor heterogeneity (**A**) Frequency plot of 8p11-p12 amplification in 47 breast tumors. The x-axis depicts the genomic position on chromosome 8 in Mb according to UCSC May 2004 hg17: NCBI Build 35 from the telomere on the 8p arm to the centromere; y-axis, percentage of tumors with amplification (green) and loss (red). Vertical dashed lines indicate the nine most common amplification peaks (P1-P9). Horizontal green lines indicate the DNA amplification regions for each tumor sample. (**B**–**C**) Zoom-in of peaks P2–P5 showing BAC clones used in locus-specific dual-color FISH and significantly associated genes spanning each peak. Biotin-labeled (green) BAC clones RP11-621B1 (P2), RP11168H8 (P3), RP11-350N15 (P4), RP11-742H5 (P5) were combined with digoxigenin-labeled (red) BAC clones RP11-527N22 (P2), CTD-2015B18 (P3), RP11-389E22 (P4), RP11-732D22 (P5). Overlapping DNA sequences were detected as yellow hybridization signals. The interphase nuclei were counterstained with DAPI.

We recently described the effect of genetic and epigenetic crosstalk in breast carcinomas harboring DNA amplification on chromosome 8p11-p12, suggesting that aberrant DNA methylation patterns on chromosome 8q may also contribute to the aggressive phenotype [12]. To further define the role that 8p11-p12 amplification may have on breast cancer pathophysiology, we examined genomic profiling data for 229 invasive breast carcinomas and transcriptomic data for 150/229 samples, as previously presented [12, 14–16]. The array-CGH copy number analysis identified 83 samples (36%) with recurrent CNAs on chromosome bands 8p11-p12 (47 high-level amplifications, 20 low-level gains and 16 losses) and 146 samples (64%) with neutral DNA dosage on chromosome 8p11-p12. Furthermore, the amplicon contained five major sub-regions mapping to a 12.0 Mb region spanning 31.9–43.9 Mb (from telomere to centromere on the 8p arm, according to the hg17 build 35 reference assembly of the human genome), which was further refined to nine minimal common amplification peaks (range, 41.2–377.4 kb) from 34.3–42.5 Mb (Figure 2A–2B and Supplementary Table 2).

One of the smallest peaks and notably the most common mapped to a 67.9 kb region spanning the *WHSC1L1* gene on chromosome band 8p12 (amplified in 32/47 cases). Dual-color interphase FISH performed using a contig of 58 overlapping BAC clones spanning the 8p11-p12 genomic region (Supplementary Table 3) demonstrated extensive intra- and intertumoral heterogeneity in amplified cases (Figure 2C). Intratumoral heterogeneity frequently ranged from neutral DNA copy number (two copies per FISH probe) to high-level amplification (up to 50 copies per FISH probe). Two main types of hybridization patterns were observed, *e.g.* hybridization signals that were clustered in set positions in the interphase nuclei or scattered signals, suggesting the presence of homogenously staining regions at 8p11-p12, translocation events with DNA sequences from chromosome 8p on other chromosomes, double minutes containing sequences from chromosome 8p and/or aneuploidy of chromosome 8.

### Allele-specific copy number analysis reveals elevated DNA copy number for one allele at the 8p11-p12 locus

**Figure 3:**
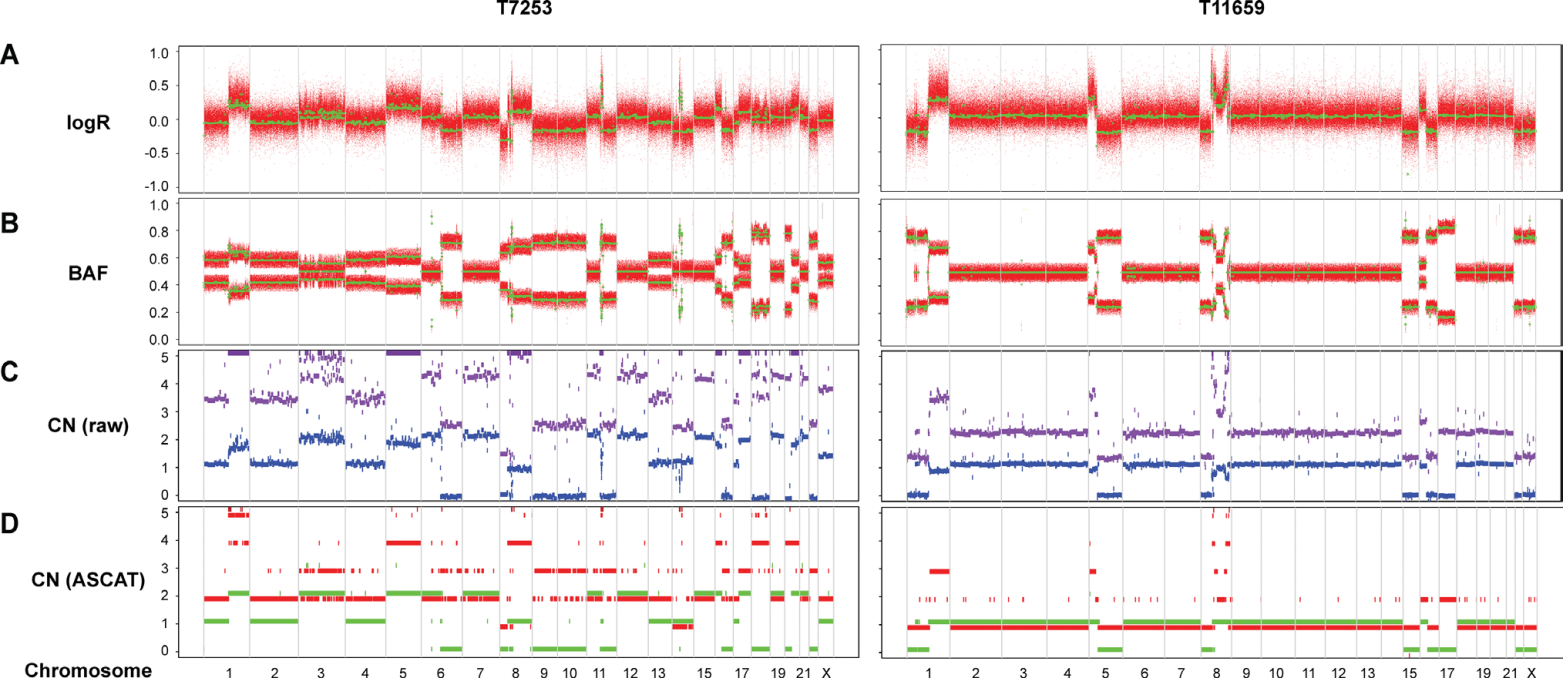
Allele-specific copy number analysis reveals elevated DNA copy number for the 8p11-p12 genomic region on one of the alleles SNP genotyping copy number profiles for samples T7253 and T11659, showing (**A**) logR and (**B**) B allele frequency (BAF) plots. Green lines indicate genomic segments of constant logR and BAF values, as identified using the ASCAT (allele-specific copy number analysis of tumors) algorithm. (**C**) Raw ASCAT profile containing allele-specific copy number for all loci. The x-axis depicts the genomic location and the DNA copy number on the y-axis. Purple and blue indicate the copy number of the minor allele and the estimated overall copy number, respectively. (**D**) ASCAT profile containing probes that are heterozygous in the germline. The x-axis depicts the genomic location and the DNA copy number on the y-axis. Green and red indicate the copy number of the minor allele and the estimated overall copy number, respectively.

**Figure 4:**
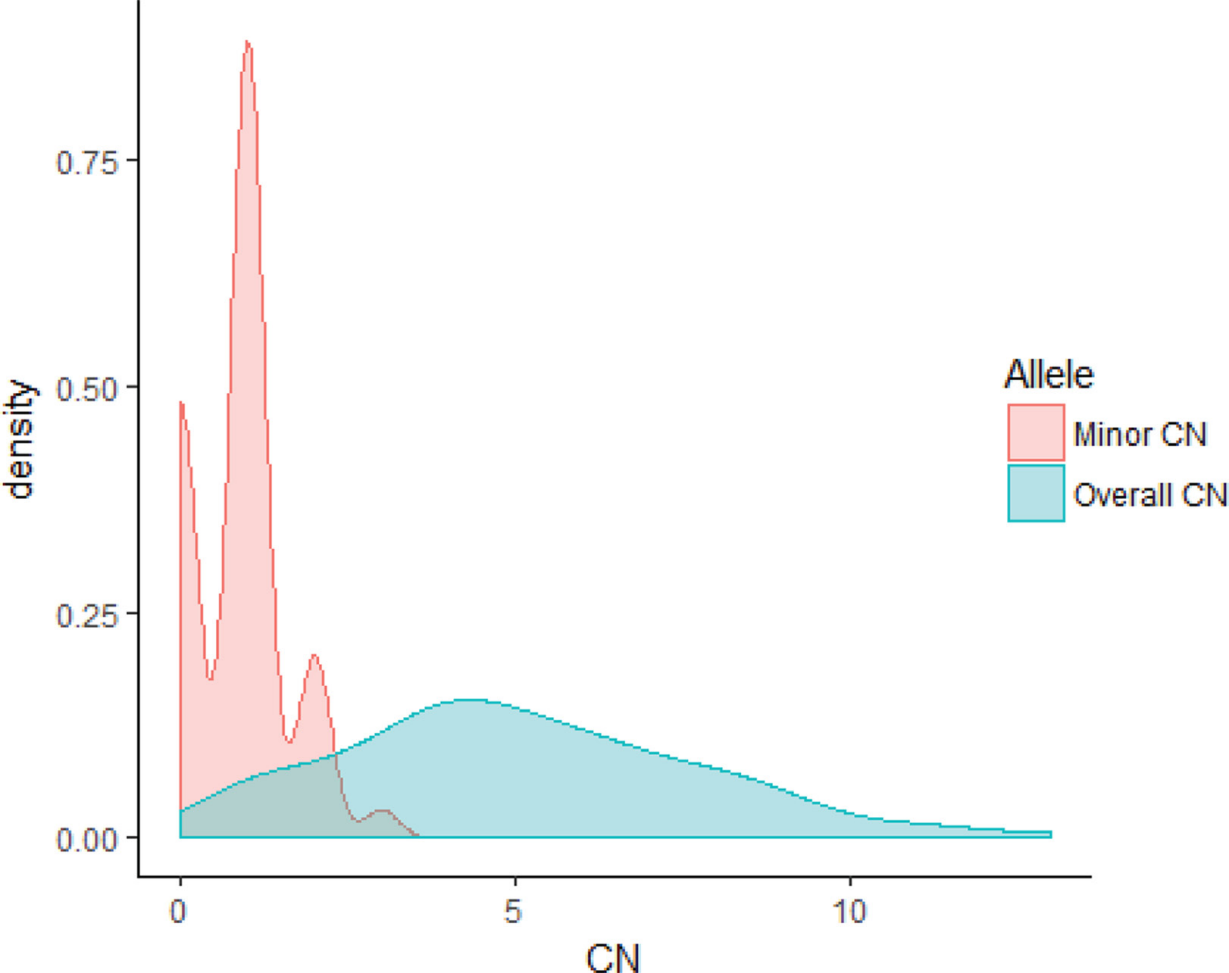
Distribution of the minor and overall copy number in the 23 samples Density plot for the minor and overall copy number spanning the 8p11-p12 genomic region (31.9–43.9 Mb). The overall copy number ranged from 1 to 13, whereas the minor allele displayed comparatively low copy number (range 0–3). The x-axis depicts the copy number; y-axis, density.

Whole-genome SNP genotyping analysis was then performed, followed by genome-wide allele-specific copy number analysis with the ASCAT algorithm for 23 samples (16 Luminal B/HER2-, two Luminal B/HER2+, four HER2/ER-, and one Basal-like subtype) harboring high-level regional 8p11-p12 amplification. High-level amplification of the 8p11-p12 genomic region was shown in the ASCAT profiles for all but one case (T7631). Furthermore, 48% of cases were classified as aneuploid (ploidy > 2.7) with on average 40% nonaberrant cell admixture. Allelic imbalances spanning the 8p11-p12 region (31.9–43.9 Mb) were detected (Pearson correlation = −0.21), where the minor allele (least frequent allele) frequently displayed CN = 0, CN = 1 or CN = 2, while the overall copy number ranged from CN = 1 to CN > 10 (Figures 3–4). These findings suggest that certain parts of chromosome 8p11-p12 were amplified on the major allele, whereas neutral DNA dosage or DNA loss were found on the minor allele.

### RNA sequencing identifies long non-coding RNAs as promiscuous fusion partners

The 23 amplified tumors were further assessed for genetic variants and structural rearrangements with RNA-seq, followed by SNP genotyping analysis to further refine the DNA breakpoints involved with the formation of fusion transcripts. In total, 3,052 fusion transcripts (1,245 unique fusion transcripts) were detected in the 23 tumors, with 237, 37, and 7 recurrent fusions found in at least two, ≥ 5, and ≥ 10 samples, respectively (Supplementary Figure 1A). The mean number of fusion transcripts identified per tumor was 132.7 ± 31.0 (± SEM, range 12–613). Few fusions (*n* = 46) contained at least one gene fusion partner that spanned the 8p11-p12 genomic region (*ADAM2*, *ADAM32*, *ADAM9*, *ASH2L*, *BAG4*, *DDHD2*, *EIF4EBP1*, *ERLIN2*, *FGFR1*, *HOOK3*, *PLPP5* (gene alias *PPAPDC1B*), *RAB11FIP1*, *TACC1*, *WHSC1L1*), of which four fusions were in-frame (*ADAM9-HOOK3, BAG4-PDSS2, NUP93-DDHD2, TACC1-EIF4EBP1).* In addition, three of the 46 fusion transcripts were found in two samples (*ERLIN2*-*MALAT1*, *MALAT1*-*TACC1*, *NUP93*-*DDHD2*), whereas the other 43 fusions were either unique to a specific tumor specimen or alternative splicing events of the same fusion transcript.

The majority of the 3,052 fusion transcripts (86%) contained at least one gene partner predicted to be in exonic regions (with no known coding DNA sequence, CDS), such as the *MALAT1* and *NEAT1* non-coding RNAs (ncRNAs). In contrast, relatively few fusion transcripts were predicted to be promoter-coding (5′UTR; 1.7%), coding-3′UTR (2.4%), in-frame/coding-coding (2.5%), out-of-frame/coding-coding (2.2%), and truncating (0%; Supplementary Figure 1B). Four recurrent in-frame fusion transcripts were each identified in two individual samples originating from the same patient, *i.e. MAST2-PRKCA, NUP93-DDHD2, TTC19-MYO1D,* and *VMP1-CEP112.* In-frame kinase fusions with therapeutic potential were also identified in eight fusion partners (*CDC42BPA, CSNK1A1, ERBB2, ERBB4, LRGUK, MAST2, PRKCA*, and *TAOK1* genes). Additionally, only one fusion transcript spanned the promoter regions of both gene partners (*TRPS1*-*CPB1*). Fifty-one percent of fusion transcripts contained gene partners with inverted orientation, implying fusion transcript formation via inversion events.

**Figure 5:**
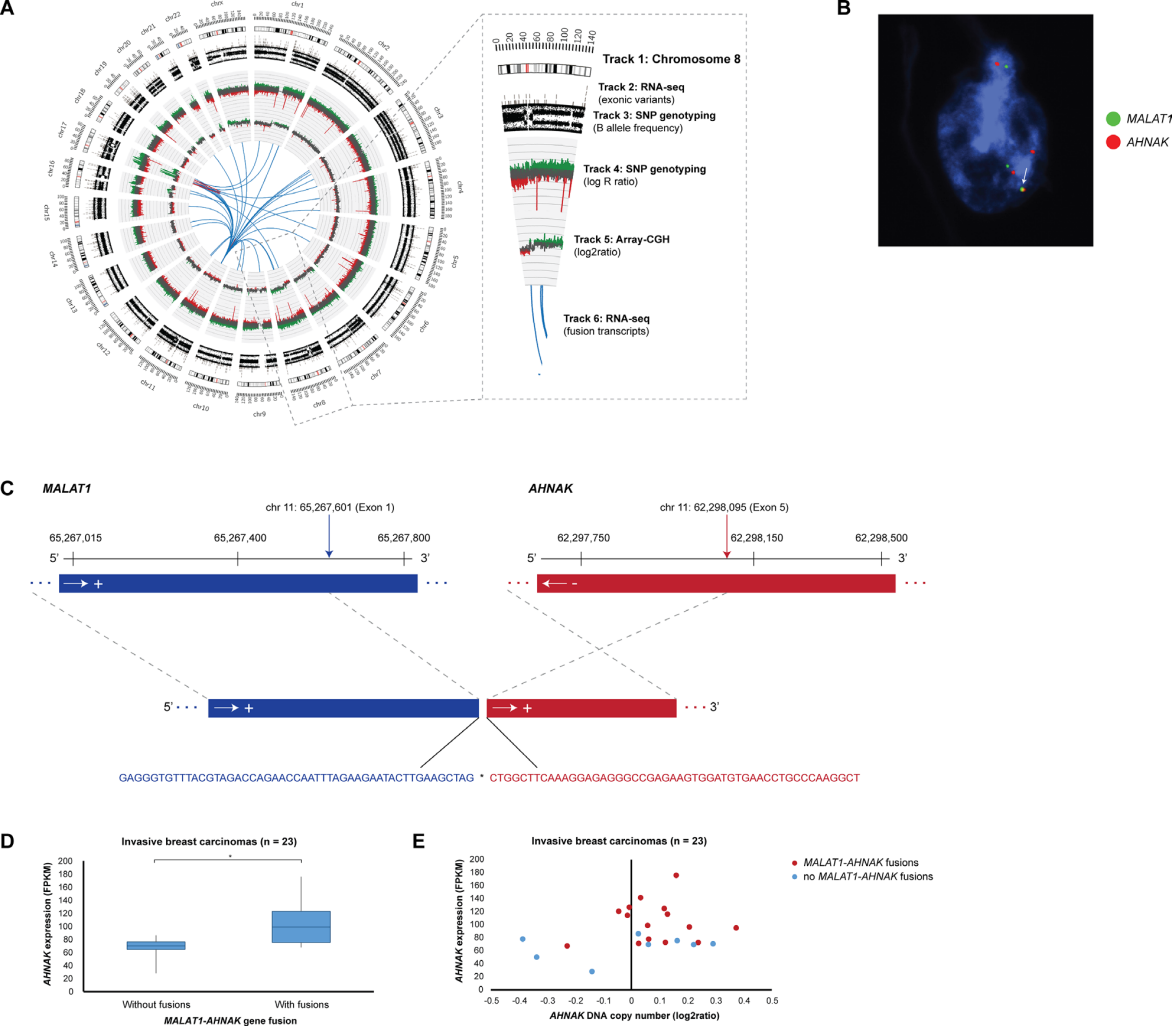
Elevated *AHNAK* expression levels in samples harboring *MALAT1-AHNAK* gene fusions (**A**) Circos plot depicting genome-wide SNP genotyping, array-CGH, and RNA-seq data in breast carcinoma sample T11378. *Track 1:* Chromosome cytobands from pter to qter. The centromere is shown as a red bar. *Track 2:* Mutations in exonic regions (exonic variants) identified with RNA-seq data are shown as dark gray bars. *Track 3:* B allele frequency of SNP genotyping data. *Track 4:* Log R ratio of SNP genotyping data, where copy number gains and losses are depicted in green and red, respectively. *Track 5:* Array-CGH data, where copy number gains and losses are depicted in green and red, respectively. *Track 6:* Gene fusions identified with RNA-seq data. Intrachromosomal and interchromosomal gene fusions are shown in red and blue lines, respectively. (**B**) Locus-specific dual-color FISH with biotin-labeled (green) BAC clones RP11-642F7, RP11-1104L6, RP11-472D15 for *MALAT1* were combined with digoxigenin-labeled (red) BAC clones CTD-2240J20 for *AHNAK*. Overlapping DNA sequences were detected as yellow hybridization signals (see arrow). The interphase nuclei were counterstained with DAPI. (**C**) Overview of the exon structure, breakpoint location, and nucleotide sequence for the *MALAT1*- *AHNAK* fusion transcript (5′ and 3′ fusion gene partners are depicted in blue and red, respectively) in sample T11378. The breakpoint sequence positions for each of the fusion gene partners are indicated by vertical arrows and the DNA strand orientation by horizontal arrows. The exon structure and nucleotide sequence at the fusion transcript breakpoint (indicated by asterisk) is indicated by dotted gray lines. Sequence positions are based on alignment with hg19 from the UCSC Genome Browser. (**D**) Breast carcinomas containing *MALAT1*-*AHNAK* gene fusions have elevated *AHNAK* expression levels (*P* = 0.0031). Statistically significant differences (*P* < 0.05) in *AHNAK* expression levels between tumors with and without *MALAT1*-*AHNAK* gene fusions are indicated by an asterisk (^*^). (**E**) Breast carcinomas containing *MALAT1*-*AHNAK* gene fusions (dark red dots) have elevated *AHNAK* expression levels compared to tumors without gene fusions (blue dots).

Among the 1,245 unique fusion transcripts, interchromosomal fusions (*n* = 1,089) were significantly more prevalent than intrachromosomal fusions (*n* = 158). In particular, chromosome 11 formed fusion transcripts with all other autosomal chromosomes and the X chromosome (*n* = 437). Intrachromosomal fusions on chromosome 11 and 8 were most common with 84 and 23 fusion transcripts, respectively (Supplementary Figure 1C–1D). The highest number of interchromosomal fusions were found on chromosomes 1–11, 11–1, 11–8 and 8–11 with 61, 52, 45, and 45 fusions in the patient cohort, respectively. Locus 11q13.1 (5′-gene partner) fused with 303 different loci followed by 11q12.3 (24 other loci), 8q23.3 (15 other loci), 2q35 (13 other loci), 3q24 (13 other loci), and 8q21.11 (11 other loci). As the 5′-gene partner, loci 8p11.25, 8p11.22, 8p11.23-p11.22, 8p11.21, 8p12 fused with 5, 4, 3, 1, and 1 other loci, respectively. The top 5′-gene partners included the *MALAT1* (11q13.1), *NEAT1* (11q13.1), *SCGB2A2* (11q12.3), and *TRPS1* (8q23.3) genes with 411, 30, 19, and 15 3′-gene partners, respectively. Additionally, the top 3′-gene partners included the *MALAT1*, *NEAT1*, *SCGB2A2*, and *TRPS1* genes with 445, 39, 20, 14 5′-gene partners, respectively. Gene reciprocals (geneA-geneB and geneB-geneA) were common among the top gene partners, suggesting inversion-mediated gene fusion formation. In the case of *MALAT1*-*AHNAK*/*AHNAK*-*MALAT1* and *MALAT1*-*TRPS1*/*TRPS1*-*MALAT1* gene reciprocal fusions, the 3′-gene partners (*AHNAK* and *TRPS1*) had significantly higher expression levels in samples containing the fusion only when *MALAT1* was the 5′-gene partner (Figure 5). FISH analysis for 12 recurrent fusion transcripts, including *AHNAK*-*MALAT1* fusions, revealed intratumoral heterogeneity with few neoplastic cells containing specific fusions on the DNA level.

A review of the array-CGH data showed that only one-third of fusion breakpoints could be attributed to DNA copy number gains and losses. However, SNP genotyping revealed that the majority of fusions occur at DNA breakpoints in addition to allelic imbalance on almost all chromosomes (Figure 5). Intrachromosomal fusions, in particular, frequently spanned regions of high-level amplification. As expected, the majority of recurrent fusions spanning genomic regions with DNA copy number changes included fusions with the *MALAT1, NEAT1,* and *TRPS1* genes, but also *NDUFC2-KCTD14-TMSB15A* (*n* = 2), *NDUFC2-TMSB15A* (*n* = 2), *FANCC-CNTNAP2* (*n* = 2), *VMP1-CEP112* (*n* = 2), *ZNF671-SPAG1* (*n* = 2).

Consequently, the Oncofuse Bayesian classifier pipeline classified 83/1,245 (6.7%, range 0–20) fusion transcripts (65 fusion genes) as “driver” fusion events with oncogenic properties, including the *COL1A2*-*TRPS1* and *MAST2*-*PRKCA* fusions that were identified in more than one tumor and several other known breast cancer-related genes, *e.g. BCL2*, *ESR1*, *ERBB2*, *IGFBP5*, *TRPS1* (Supplementary Table 4). One or both of the gene fusion partners (27/65 and 19/65 fusion transcripts, respectively) also frequently exhibited high gene expression patterns irrespective of DNA copy number in samples harboring “driver” fusion events (Supplementary Figure 2). Pathway analysis showed that these fusion transcripts play a pivotal role in cancer, cell cycle, DNA replication, recombination and repair, cell death and survival, cellular growth and proliferation, cellular movement, ErbB signaling, PTEN signaling, and DNA double-strand break repair by homologous recombination (*P* < 0.05).

### Mutation analysis reveals few amplification-specific exonic variants

The RNA-seq data were then evaluated to identify recurrent insertions/deletions (indels) and single-nucleotide variants (SNVs) in genomic and exonic (coding) regions. The dbSNP, 1000 Genomes Project, SweGen dataset, and NHLBI GO Exome Sequencing Project databases were used to identify and remove common genetic variants present in the human population. After filtering, the mean number of genomic and exonic variants per tumor was 85,066 ± 4,127.9 (± SEM; range, 46,290–115,402) and 399.0 ± 16.2 (range, 279–540), respectively. Genomic variants were most commonly identified in the intronic regions (mean, 60,496), in addition to nonsynonymous SNVs (mean, 211.0) and synonymous SNVs (mean, 85.0) in the coding regions (Supplementary Figure 3A–3B). Single-nucleotide substitutions associated with A > G (44.0%) and T > C transitions (43.4%) were most prevalent in the genomic regions and A > G (16.9%), T > C (16.2%), G > A (15.7%), and C > T transitions (14.6%) in coding regions (Supplementary Figure 3C–3D). In addition, 11 tumor-specific exonic variants were identified in six genes spanning the 8p11-p12 genomic region (*RAB11FIP1, GPR124, ADAM2, LSM1, TACC1, ZNF703*).

**Figure 6:**
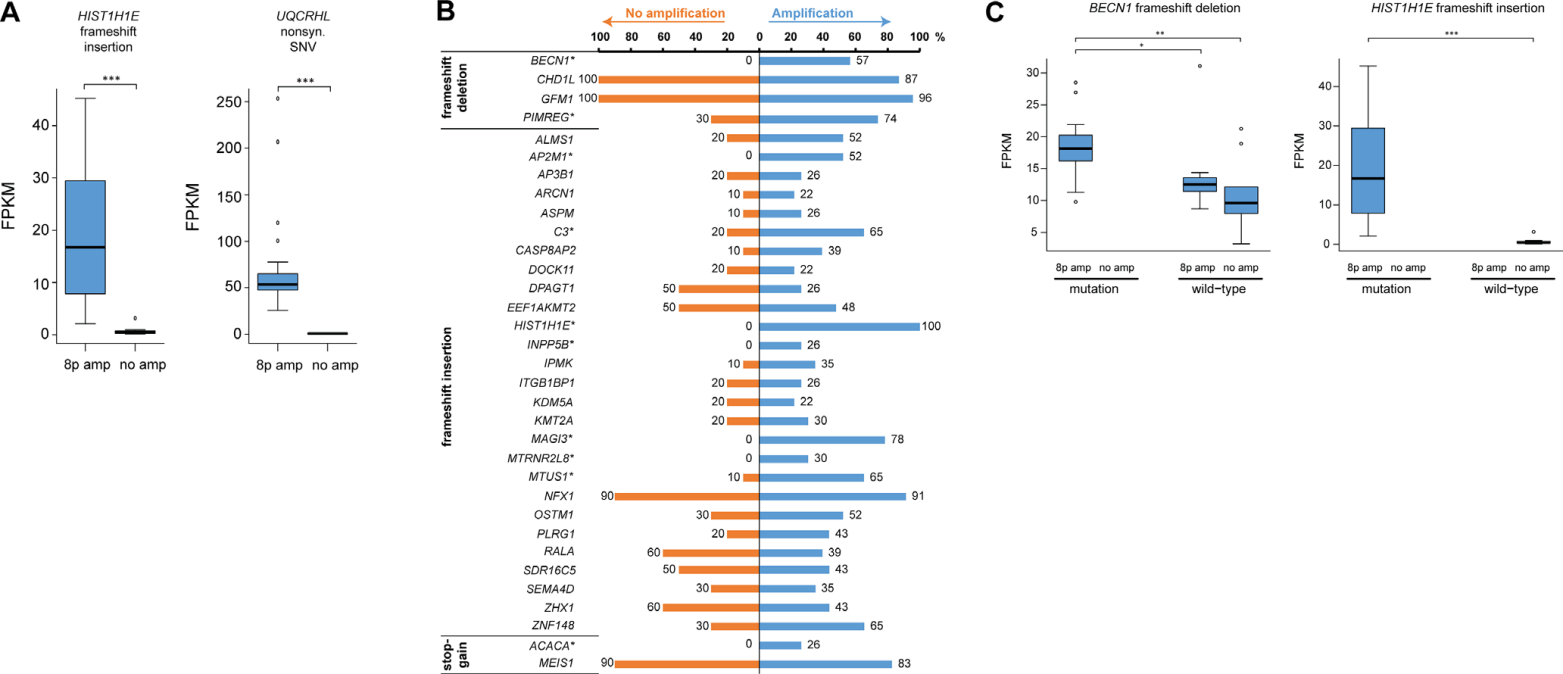
Few exonic variants are associated with 8p11-p12 amplification (**A**) Box plots illustrating mutation-dependent changes in FPKM values for two genetic variants found in all 23 samples with 8p11-p12 amplification (denoted *8p amp*) and none of the TCGA breast carcinoma samples with neutral 8p11-p12 copy number (denoted *no amp*). (**B**) Bar plot illustrating the percentage of 8p11-p12 amplified samples (blue bars) and TCGA breast carcinoma samples with neutral 8p11-p12 copy number (orange bars) harboring a putative deleterious genetic variant in exonic regions (in at least 20% of amplified samples). Genetic variants with significantly different mutation frequencies in the two groups were marked with an asterisk (^*^) symbol (*P* < 0.05). (**C**) Box plots illustrating the effect of 8p11-p12 amplification and mutation status on FPKM values. Asterisk denotes significant *p*-values (^*^P < 0.05, ^**^P < 0.01, ^***^P < 0.001).

The mutational landscape was also assessed in 10 non-amplified breast carcinomas from The Cancer Genome Atlas (TCGA). As expected, the mean number of genomic variants was significantly lower in the non-amplified TCGA samples (10,822.2 ± 1,113.0; range, 4,073–15,688) than the 8p11-p12 amplified tumors due to the use of whole transcriptome sequencing in the current investigation and mRNA-seq for the TCGA dataset. However, there was no significant difference in the mean number of exonic variants (358.7 ± 43.1; range, 216–677, in the TCGA cohort), the type of exonic variants or single-nucleotide substitutions identified in the two study groups (Supplementary Figure 3B and 3D). The distribution of indels and SNVs in coding regions was also evaluated in the non-amplified samples to identify exonic variants associated with 8p11-p12 amplification. Frameshift insertion in *HIST1H1E* (encoding p.Ala167fs) and nonsynonymous SNV in *UQCRHL* (encoding p.His56Arg) were only present in samples harboring 8p11-p12 amplification and resulted in mutation-dependent changes in gene expression levels. Consequently, neither of the two transcripts have been previously reported in the Catalogue Of Somatic Mutations In Cancer (COSMIC) database (Figure 6A).

Sequence Ontology analysis was then performed to identify potential deleterious genetic variants predicted to have a disruptive effect in the protein by resulting in protein truncation, gain/loss of function or nonsense mediated decay, *i.e.* frameshift insertion, frameshift deletion, frameshift block substitution, stopgain, or stoploss. In total, 33 potential deleterious genetic variants were identified in ≥ 20% of the amplified tumors; none of the 33 genetic variants were found in the COSMIC database (Figure 6B). Pathway analysis showed that the genes associated with the genetic variants play a pivotal role in cancer, cell cycle, cell death and survival, cell morphology, and gene expression. To distinguish whether the 33 deleterious genetic variants were 8p11-p12 amplification-specific, the mutation frequency was then evaluated in the 10 non-amplified TCGA samples. This analysis showed that 10/33 genetic variants (*ACACA*, *AP2M1, BECN1, C3, HIST1H1E, INPP5B, MAGI3, MTRNR2L8, MTUS1, PIMREG*) had significantly higher mutation rates in 8p11-p12 amplified samples, of which 7/10 genetic variants were only found in amplified samples (*P* < 0.05). The 10 genetic variants were further evaluated to assess the effect of 8p11-p12 amplification status and/or mutation on gene expression patterns. Consequently, altered gene expression levels for all 10 genetic variants (*ACACA*, *AP2M1, BECN1, C3, HIST1H1E, INPP5B, MAGI3, MTRNR2L8, MTUS1, PIMREG*) were dependent on amplification status (8p11-p12 amplified versus non-amplified samples). Three genetic variants (*BECN1* frameshift deletion, *MTUS1* frameshift insertion*, PIMREG* frameshift deletion) showed mutation-dependent (mutated versus wild-type samples) changes in gene expression in 8p11-p12 amplified samples (*P* < 0.05; Figure 6C).

## DISCUSSION

We report that few genes spanning the 8p11-p12 amplicon in breast carcinoma are involved in genetic mutations and DNA methylation modifications, suggesting that DNA amplification is the primary mode of gene activation for this genomic region [12, 17–19]. In this study, an integrative analysis with multi-omics screening identified previously unknown recurrent genetic features, ranging from chromothripsis events to fusion transcripts, associated with 8p11-p12 amplification in breast carcinoma. Using array-CGH and SNP genotyping data, we illustrated that invasive breast tumors frequently contain complex rearrangements on one or two chromosomes (chromothripsis-like patterns) spanning regions of DNA amplification, including the 8p11-p12 genomic region. Further examination of the 8p11-p12 amplicon showed that DNA amplification was restricted to only one of the alleles, indicating allele-specific amplification events.

DNA copy number analysis revealed recurrent chromothripsis-like events spanning the 8p11-p12 amplicon, including an amplification peak comprised of the histone lysine methyltransferase *WHSC1L1* (Wolf-Hirschhorn syndrome candidate 1-like 1), also known as *NSD3*. *WHSC1L1* has been studied extensively to better understand its role in 8p11-p12 amplification in breast carcinoma and other malignancies [17, 20–23]. At least two co-expressed WHSC1L1 isoforms (the long and short isoforms) compete for binding sites on target proteins [24]. The full-length WHSC1L1 protein contains several functional domains with methyltransferase and protein binding activity, which play a pivotal role in chromatin modification and regulation of transcription by methylating lysine-27 of histone H3 (epigenetic tag denoting inhibition of transcription). In contrast, the short isoform contains a single PWWP-domain (proline-tryptophan-tryptophan-proline) that may be involved in cell growth. In the absence of 8p11-p12 amplification, Zhou *et al.* showed an increase in cell proliferation and cell invasion in the MDA-MB-231 breast cancer cell line following *WHSC1L1*-long knockdown [24, 25]. These findings are in contrast with results found in breast cancer cell lines harboring the 8p11-p12 amplicon, where cell proliferation decreased after *WHSC1L1* depletion [23]. In addition, it is still unclear whether *WHSC1L1* overexpression really does play a role in cell cycle regulation of G2/M transition by activating *CCNG1* and *NEK7* [26, 27]. WHSC1L1 was also found to regulate methylation of lysine-36 on histone 3 and transcriptional elongation by binding to LSD2 (a H3K4-specific lysine demethylase), and G9a (a H3K9-specific methyltransferase) [28–31]. Although recent reports have shown that protein methyltransferases can be targeted with small-molecule inhibitors, none are currently used in clinical practice [26, 32, 33].

Whole-transcriptome RNA-seq and genome-wide SNP genotyping analysis highlighted the prevalence of fusion transcripts and genetic variants in 8p11-p12 amplified tumors. These analyses revealed that almost 90% of the identified fusion gene partners were ncRNAs, such as *MALAT1* (metastasis-associated lung adenocarcinoma transcript 1; also known as *NEAT2*) and *NEAT1*. *MALAT1* was highly promiscuous with over 400 gene partners (as both the 5′- and 3′-gene partner), suggesting that these fusions occur at the RNA level. *MALAT1* is an evolutionary conserved gene that has been shown to be involved in chromosomal translocations and contain genetic variants [34, 35]. Like other ncRNAs, *MALAT1* can migrate from the nucleus to the cytoplasm where it can interact with both DNA and proteins in the nucleus and cytoplasmic RNA molecules and proteins [36]. *MALAT1* is particularly interesting because it is a) frequently overexpressed in different malignancies, b) a prognostic indicator of poor survival in breast cancer, c) has been shown to be controlled by 17β-estradiol stimulation in prostate cancer, d) *c-MYC* has been shown to bind to the *MALAT1* promoter thereby inducing *MALAT1* transcription, and e) has been shown to be associated with cell proliferation, metastasis, and the cell cycle [37–41]. Furthermore, a *Malat1* knockout mouse model resulted in normal pre- and postnatal development and *Malat1* inhibition in a mouse model for luminal B breast cancer gave rise to poorly developed metastatic tumors, suggesting that MALAT1 inhibition may be a feasible approach to reduce tumor growth and metastasis with minimal adverse effects on normal tissue [40, 41].

Among protein-coding genes, several breast cancer-related genes were predicted to be fusion transcripts with oncogenic potential, *e.g. BCL2*, *ESR1*, *ERBB2*, *IGFBP5*, *TRPS1*. Interestingly, we have previously shown that *TRPS1* is among other genes spanning chromosome 8q that are hypomethylated in 8p11-p12 breast tumors [12]. Fusions are commonly produced during the formation of structural rearrangements, transcription read-throughs, and alternative splicing, where one fusion partner frequently deregulates the other [42]. As expected, the majority of the fusion transcripts identified here spanned genetically instable regions with DNA breakpoints, particularly intrachromosomal fusions. It was also shown that in recurrent fusions, such as *MALAT1*-*AHNAK*/*AHNAK*-*MALAT1* and *MALAT1*-*TRPS1*/*TRPS1*-*MALAT1,* our data suggest that *MALAT1* only deregulated the expression patterns of its gene partner (*AHNAK* or *TRPS1*) when *MALAT1* was the 5′-gene partner. Additionally, several interesting in-frame fusions and in-frame kinase fusions were identified, several of which may be targetable with kinase inhibitors.

In contrast to the fusion transcripts, few ncRNAs contained genetic variants such as indels and substitutions. Intriguingly, genetic variants in *HIST1H1E* encoding p.Ala167fs (frameshift insertion) and *UQCRHL* encoding p.His56Arg (nonsynonymous SNV) were found in all 23 amplified samples and in none of the non-amplified TCGA samples. These genetic variants also resulted in significant up-regulation of the two genes in mutated/amplified samples. *HIST1H1E* is a linker histone gene that may play a role in epigenetic regulation, whereas *UQCRHL* has been identified as a prognostic factor for hepatocellular carcinoma that plays a pivotal role in mitochondrial respiration [43–45]. The few exonic variants and fusion transcripts identified in 8p11-p12 genes were tumor-specific rather than amplification-specific, suggesting these molecular mechanisms may be secondary modes of gene activation. Three exonic variants were identified in the *RAB11FIP1* and *ZNF703* genes and *FGFR1*, *RAB11FIP1* and *WHSC1L1* were among fourteen genes spanning the 8p11-p12 amplicon to be identified as fusion gene partners.

In summary, we describe the genetic landscape of 8p11-p12 amplification in breast carcinoma, including previously undescribed chromosomal rearrangements and gene fusions. Our work may pave the way for future studies investigating the mechanisms by which specific oncogenes within the 8p11-p12 amplification region promote breast tumorigenesis, which may lead to more specific target therapies and thereby improve treatment for patients with 8p11-p12 amplified breast carcinomas.

## MATERIALS AND METHODS

### Evaluation of genomic and transcriptomic profiling data

To further investigate the clinical significance of 8p11-p12 DNA amplification in breast carcinomas, genomic profiling data for 229 primary invasive breast carcinomas (corresponding to 185 patients) previously profiled with microarray-based comparative genomic hybridization (array-CGH) and gene expression microarray data for 150 of the 229 samples (corresponding to 140 patients) [12, 14–16] were evaluated and correlated with clinicopathological features and clinical outcome. Normalized values from five normal breast samples profiled with Illumina HumanWG-6 Expression Beadchips (GEO, accession number GSE17072) were used as normal controls [46]. The patients were diagnosed in Western Sweden between 1988 and 1999 and the fresh-frozen tumor samples were stored in the tumor biobank at the Sahlgrenska University Hospital Oncology Lab (Gothenburg, Sweden).

In brief, CNAs were defined as log_2_ratio + 0.2, ≥ + 0.5, −0.2, and ≤ −1.0 for low-level gain, high-level gain/amplification, heterozygous loss, and homozygous deletions (henceforth referred to as gain, amplification, loss and deletion) using the Rank Segmentation algorithm with Nexus Copy Number Professional 4.1 software (BioDiscovery), respectively. Minimal common regions of high-level amplification were identified when observed in at least 10% of the tumor samples using *P* < 0.01. Furthermore, the “Peaks only” setting was used to refine the common regions to amplification peaks. The dataset was stratified into the molecular breast cancer subtypes (normal-like, basal-like, luminal subtype A, luminal subtype B and human epidermal growth factor receptor 2/estrogen receptor-negative (HER2/ER-)) using gene expression microarray data (*n* = 150) as previously described [47–49]. Luminal subtype B was further stratified using array-CGH to determine the HER2 amplification status for each tumor, where HER2-positive was set to log_2_ratio ≥ + 0.5 and HER2-negative was set to log_2_ratio < −0.5 [50]. Luminal subtype B/HER2- was most prevalent (*n* = 101), followed by HER2/ER− (*n* = 18), Basal-like (*n* = 16), Luminal subtype B/HER2+ (*n* = 13), and luminal subtype A (*n* = 2). Amplified samples were predominantly classified as Luminal subtype B/HER2- (*n* = 30), followed by Basal-like (*n* = 6), HER2/ER- (*n* = 5), and Luminal subtype B/HER2+ (*n* = 4).

### Chromothripsis-like pattern (CTLP) detection

The array-CGH data were segmented using the DNAcopy package (version 1.48.0) in R/Bioconductor (version 3.3.2), followed by chromothripsis-like pattern detection using the web-based CTLPScanner (http://cgma.scu.edu.cn/CTLPScanner/) with the default settings (Genome assembly: NCBI35/hg17; Copy number status change times: ≥ 20; Log10 of likelihood ratio ≥ 8; Minimum segment size (Kb): 10; Signal distance between adjacent segments: 0.3; Genomic gains ≥ 0.3; Genomic losses ≤−0.3 [13].

### Nucleic acid isolation and purification

For SNP genotyping and RNA sequencing (RNA-seq) analysis, genomic DNA and total RNA were isolated from 10–20 mg sections of fresh-frozen tumor specimens for 23/47 samples with focal 8p11-p12 amplification. Prior to nucleic acid isolation, each specimen was evaluated for neoplastic cell content using touch preparation imprints stained with May-Grünwald Giemsa (Chemicon). Highly representative specimens with at least 70% neoplastic cell content were included in downstream analyses. Genomic DNA was isolated using the Wizard Genomic DNA extraction kit (Promega), including proteinase K treatment (Roche) followed by phenol-chloroform purification (Sigma). Total RNA was isolated with the RNeasy Lipid Tissue Mini Kit (Qiagen) according to the manufacturer’s instructions. DNA and RNA concentration were measured using Nanodrop ND-1000 (Nanodrop Technologies). The total RNA concentration was also evaluated using QuBit (ThermoFisher Scientific). RNA integrity was assessed using the RNA 6000 Nano LabChip Kit with Agilent 2100 Bioanalyzer (Agilent Technologies).

### Whole transcriptome RNA sequencing (RNA-seq)

Total RNA samples from 23 breast carcinomas with high-level regional 8p11-p12 amplification were processed at the Science for Life Laboratory (National Genomics Infrastructure Stockholm). Illumina TruSeq strand-specific RNA libraries (Ribosomal depletion using RiboZero human) containing 125 bp pair-end reads were obtained for each sample on a HiSeq2000 sequencer (Illumina). The computations were performed on resources provided by SNIC through Uppsala Multidisciplinary Center for Advanced Computational Science (UPPMAX) under Project b2015076, as described in the Supplementary Methods [51].

### Quality control

Quality control of raw RNA-seq reads was performed prior to assembly using FastQC (0.11.5). The RNA-seq reads were then trimmed and filtered with TrimGalore (0.3.3) to remove adapter sequences and reads with Phred quality scores below 20, followed by alignment to the hg19 build 37 reference assembly of the human genome using STAR (2.5.1b) [52]. Read alignment yielded approximately 40–50 million aligned reads per sample. Counts and Fragments Per Kilobase of transcript per Million mapped reads (FPKM) were calculated using HtSeq (0.6.1) [53] and Cufflinks (2.2.1) [54], respectively. Quality control statistics for mapped reads (*e.g.* gene body coverage and read distribution) were obtained using RSeQC (2.3.6).

#### Fusion gene identification

Fusion transcripts were identified with FusionCatcher (0.99.5a) using criteria to remove false positive candidate fusion events, followed by classification of “driver” fusion events (Bayesian probability scores < 0.5) with oncogenic potential using Oncofuse (1.1.1) [55, 56]. Circos plots were generated with the Circos module (0.66) to visualize DNA copy number alterations, SNP plots, fusion genes, and exonic variants for each sample [57]. The difference in gene expression patterns for specific fusion transcripts was determined using *t*-test or ANOVA, as appropriate (*P* < 0.05).

#### Variant calling and filtering

The Genome Analysis Toolkit (GATK 3.5.0) variant calling pipeline [58] and the ANNOVAR tool (2016.05.11) were used to identify and annotate genetic variants, *e.g.* SNPs and indels, in individual samples with the SplitNCigarReads, BaseRecalibrator (with dbSNP Build 138 hg19), HaplotypeCaller, and VariantFiltration (Fisher Strand (FS > 30.0) and Qual By Depth values (QD < 2.0)) tools, respectively. Common genetic variants found in the human population were removed with ANNOVAR using the dbSNP (hg19_snp138), 1000 Genomes Project (1000g2015aug) with a minor allele frequency (MAF) threshold of 0.01, SweGen dataset [59], and NHLBI GO Exome Sequencing Project (hg19_esp6500siv2_al) databases. Genetic variants not found in the COSMIC database version 70 (cosmic70) were denoted as “novel” genetic variants. Sequence Ontology analysis was performed to identify a conservative set of potential deleterious genetic variants resulting in amino acid changes, *i.e.* frameshift insertion (SO:0001909), frameshift deletion (SO:0001910), frameshift block substitution (SO:0001589), stopgain (SO:0001587), or stoploss (SO:0001578) [60]. To determine whether the deleterious genetic variants were associated with 8p11-p12 amplification, the mutation frequency was also evaluated in mRNA-seq data for 10 primary breast carcinomas sequenced by The Cancer Genome Atlas (TCGA) that lacked 8p11-p12 amplification (SNP segmented mean < 0.4) [61, 62]. BAM files for the 10 TCGA samples were downloaded from the Genomic Data Commons (GDC) Portal, converted to FASTQ format with BEDTools BAMTOFASTQ (2.25.0), and compressed with Gzip before running the GATK variant calling pipeline with RNA-seq reads aligned to the hg19 build 37 reference assembly.

### Genome-wide SNP genotyping analysis

Genome-wide SNP genotyping analysis was processed for the 23 amplified samples with Illumina Infinium HumanOmni 2.5-8 v1.3 Beadchips at the SCIBLU Genomics DNA Microarray Resource Center (SCIBLU), Department of Oncology, Lund University. The beadchips were scanned on an iScan (Illumina) and data processed using the Illumina GenomeStudio Genotyping Module software (V2011.1) and hg19 build 37 reference assembly of the human genome to calculate B-allele frequencies (BAF) and logR ratios (LRR). Genome-wide allele-specific copy number profiles were generated in R/Bioconductor (version 3.3.2) using the ASCAT (allele-specific copy number analysis of tumors, version 2.5) algorithm and the germline genotype prediction function for Illumina 2.5M SNP arrays, as previously described [63]. ASCAT profiles illustrate the copy number for the minor allele (least frequent allele) and the estimated overall copy number (sum of the minor and major allele counts).

### Fluorescence *in situ* hybridization (FISH)

Probe labeling and hybridization were done using locus-specific bacterial artificial chromosome (BAC; BACPAC Resources Center) probes to verify gene amplification and fusion genes. Touchprint preparations were prepared with fresh-frozen tumor samples on Superfrost Plus microscope slides (Erie Scientific Company). Dual-color FISH was performed using co-hybridized biotin-16-dUTP and dioxigenin-11-dUTP labeled probes (Supplementary Table 3). The slides were analyzed using a Leica DMRA2 fluorescent microscope (Leica) equipped with an ORCA Hamamatsu CCD (charged-couple devices) camera and filter cubes specific for green fluorescein isothiocyanate (FITC), red rhodamine, and UV for DAPI visualization. Digitalized black and white images were acquired using the Leica CW4000 software package.

### Ingenuity pathway analysis (IPA)

Ingenuity Pathway analysis (Ingenuity Systems, Redwood City, USA) was performed to assess the functional relevance of the differentially expressed transcripts, deleterious genetic variants (identified in ≥ 20% of samples), and fusion genes with oncogenic potential. Canonical pathways, diseases and bio functions, and upstream regulator analyses were generated using Fisher’s exact test (*P* < 0.05). The activation state of the upstream regulators was determined with the z-score, where z > 2 and z < −2 were denoted as activation and inhibition, respectively.

### Statistical analyses

Statistical analyses were performed using a 0.05 *p*-value cutoff in R/Bioconductor (version 3.3.2). All *p*-values are two-sided. The difference in mutation frequency and gene expression patterns between 8p11-p12 amplified and non-amplified samples were determined using Wilcoxon Rank Sum test or Pairwise Wilcoxon Rank Sum Test.

### Data availability

The data reported in this study have been deposited in the NCBI Gene Expression Omnibus and are accessible through GEO Series accession number GSE97293 (https://www.ncbi.nlm.nih.gov/geo/query/acc.cgi?acc=GSE97293).

## SUPPLEMENTARY MATERIALS FIGURES AND TABLES


