# Supplementary material for: Genome-wide multi-omics profiling of the 8p11-p12 amplicon in breast carcinoma
Source: Oncotarget. 2018 May 8;9(35):24140–54. doi: 10.18632/oncotarget.25329 (PMC5963621; doi:10.18632/oncotarget.25329)
Supplement: Supplementary file 1 [file oncotarget-09-24140-s001.pdf]

Genome-wide multi-omics profiling of the 8p11-p12 amplicon in breast carcinoma

SUPPLEMENTARY MATERIALS

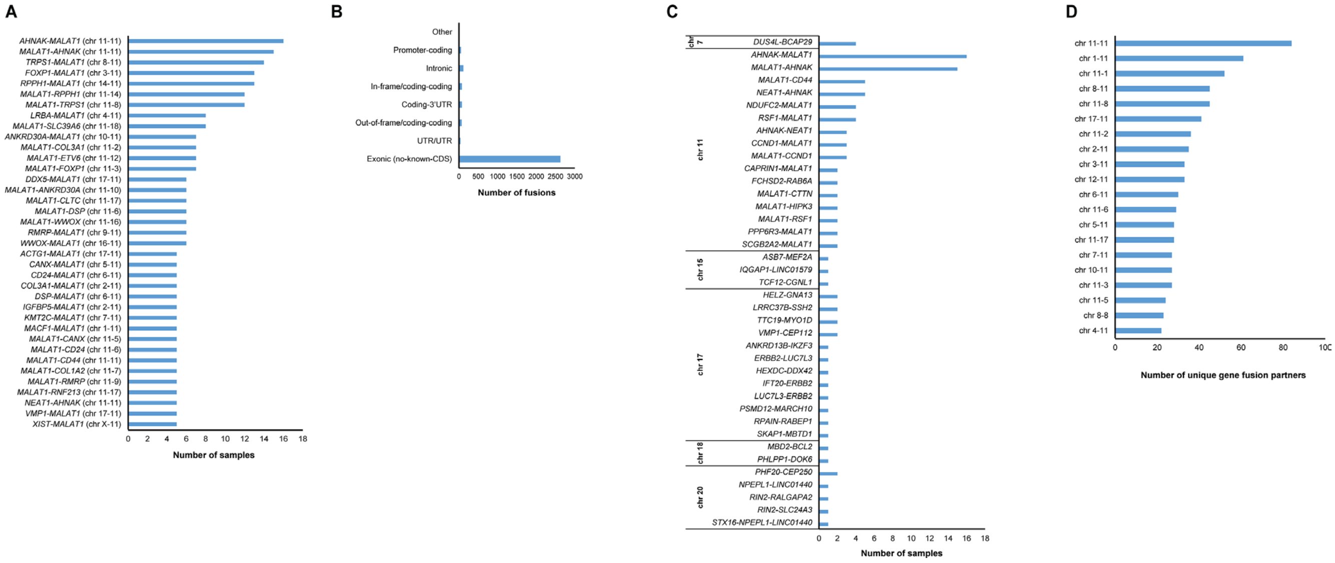

Supplementary Figure 1: The *MALAT1* ncRNA is a promiscuous gene fusion partner in 8p11-p12 amplified breast carcinomas. Bar plots depicting (**A**) recurrent fusion transcripts found in at least five samples, (**B**) the distribution of the predicted effect of the 3,052 identified fusion transcripts, (**C**) intrachromosomal fusions, and (**D**) the number of unique gene fusion partners in the top 20 chromosome (chr) pairs.

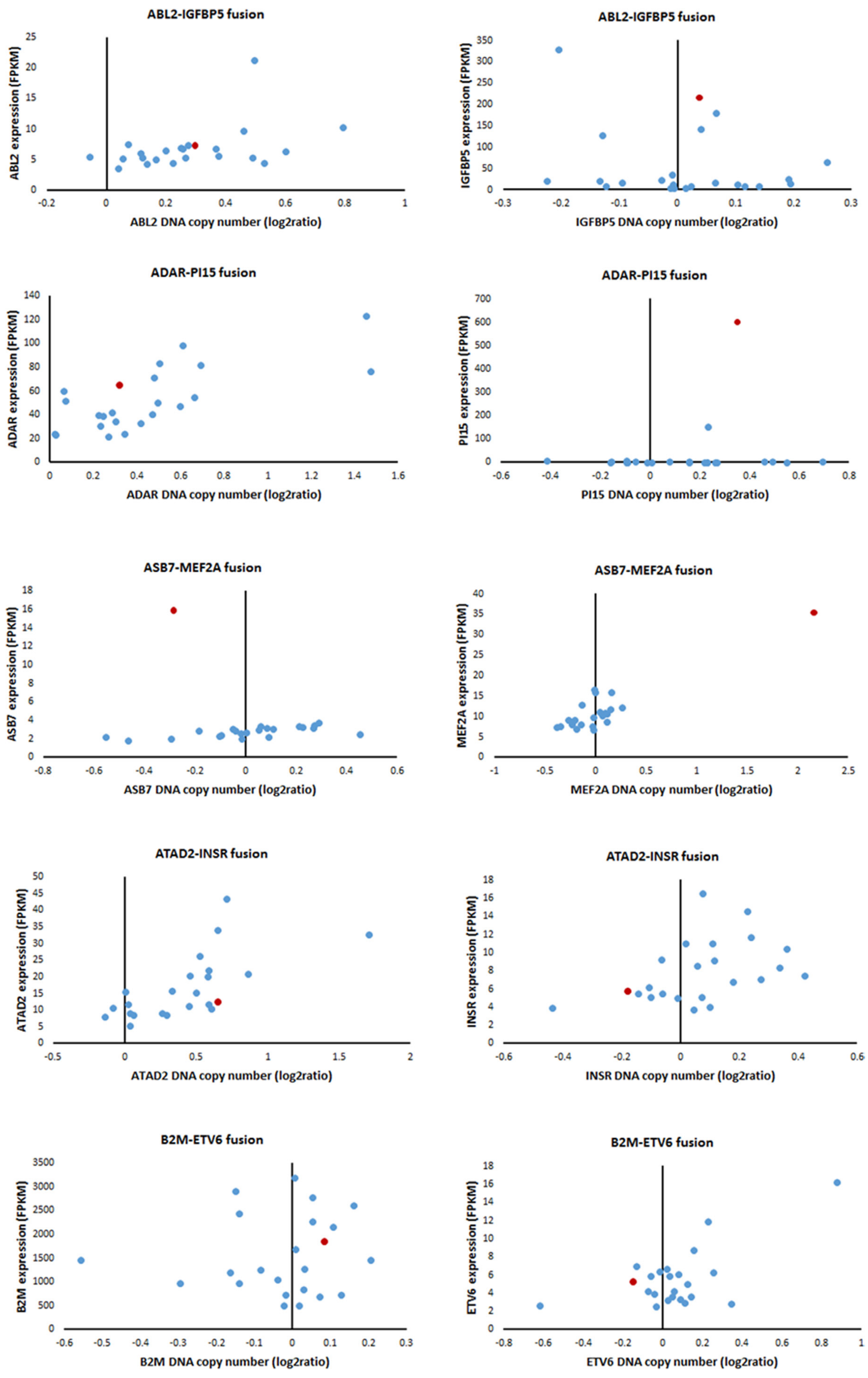
ABL2-IGFBP5 fusion
ABL2 expression (FPKM)
ABL2 DNA copy number (log2ratio)
ABL2-IGFBP5 fusion
IGFBP5 expression (FPKM)
IGFBP5 DNA copy number (log2ratio)
ADAR-PI15 fusion
ADAR expression (FPKM)
ADAR DNA copy number (log2ratio)
ADAR-PI15 fusion
PI15 expression (FPKM)
PI15 DNA copy number (log2ratio)
ASB7-MEF2A fusion
ASB7 expression (FPKM)
ASB7 DNA copy number (log2ratio)
ASB7-MEF2A fusion
MEF2A expression (FPKM)
MEF2A DNA copy number (log2ratio)
ATAD2-INSR fusion
ATAD2 expression (FPKM)
ATAD2 DNA copy number (log2ratio)
ATAD2-INSR fusion
INSR expression (FPKM)
INSR DNA copy number (log2ratio)
B2M-ETV6 fusion
B2M expression (FPKM)
B2M DNA copy number (log2ratio)
B2M-ETV6 fusion
ETV6 expression (FPKM)
ETV6 DNA copy number (log2ratio)

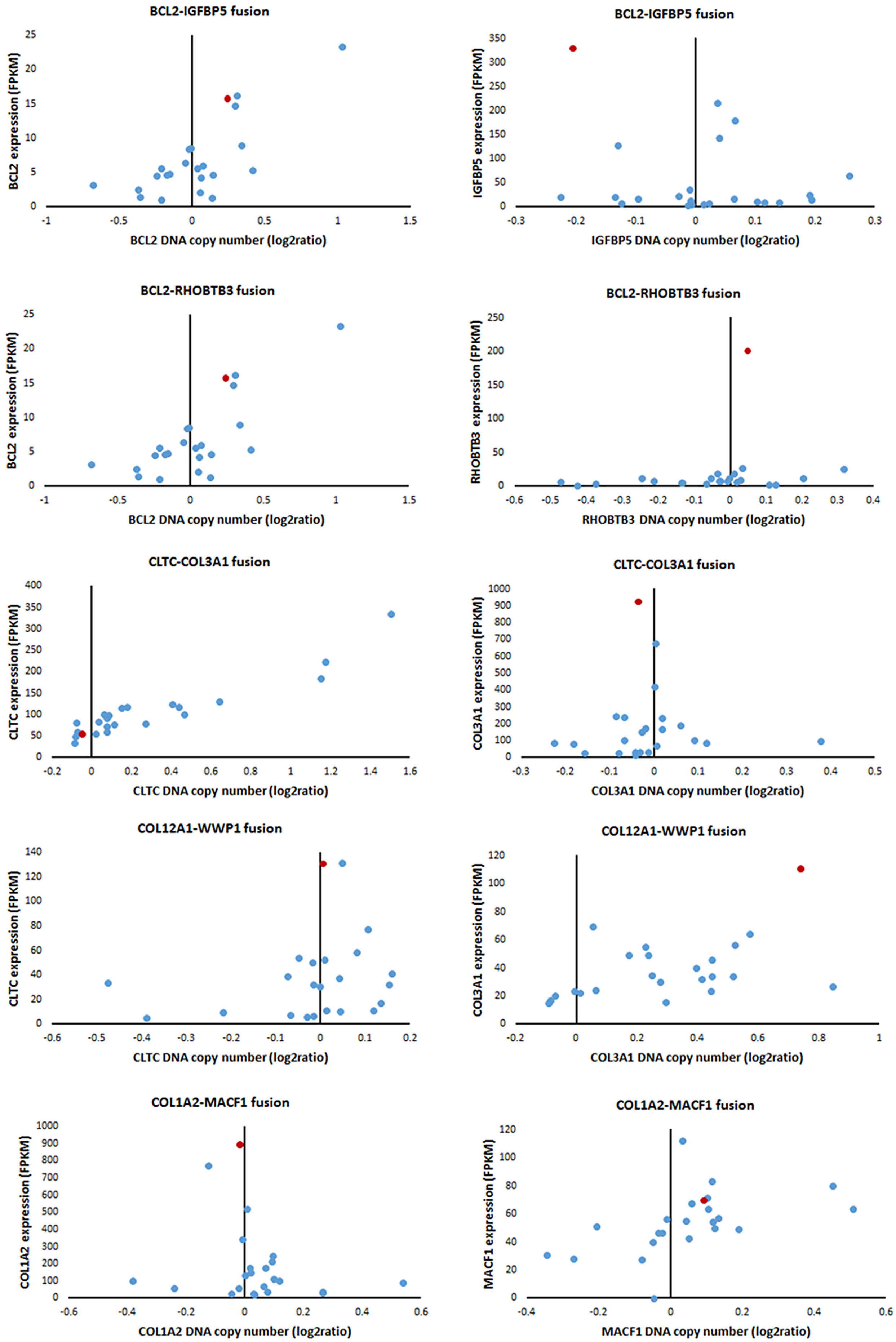

BCL2-IGFBP5 fusion
BCL2 expression (FPKM)
BCL2 DNA copy number (log2ratio)
BCL2-IGFBP5 fusion
IGFBP5 expression (FPKM)
IGFBP5 DNA copy number (log2ratio)
BCL2-RHOBTB3 fusion
BCL2 expression (FPKM)
BCL2 DNA copy number (log2ratio)
BCL2-RHOBTB3 fusion
RHOBTB3 expression (FPKM)
RHOBTB3 DNA copy number (log2ratio)
CLTC-COL3A1 fusion
CLTC expression (FPKM)
CLTC DNA copy number (log2ratio)
CLTC-COL3A1 fusion
COL3A1 expression (FPKM)
COL3A1 DNA copy number (log2ratio)
COL12A1-WWP1 fusion
CLTC expression (FPKM)
CLTC DNA copy number (log2ratio)
COL12A1-WWP1 fusion
COL3A1 expression (FPKM)
COL3A1 DNA copy number (log2ratio)
COL1A2-MACF1 fusion
COL1A2 expression (FPKM)
COL1A2 DNA copy number (log2ratio)
COL1A2-MACF1 fusion
MACF1 expression (FPKM)
MACF1 DNA copy number (log2ratio)

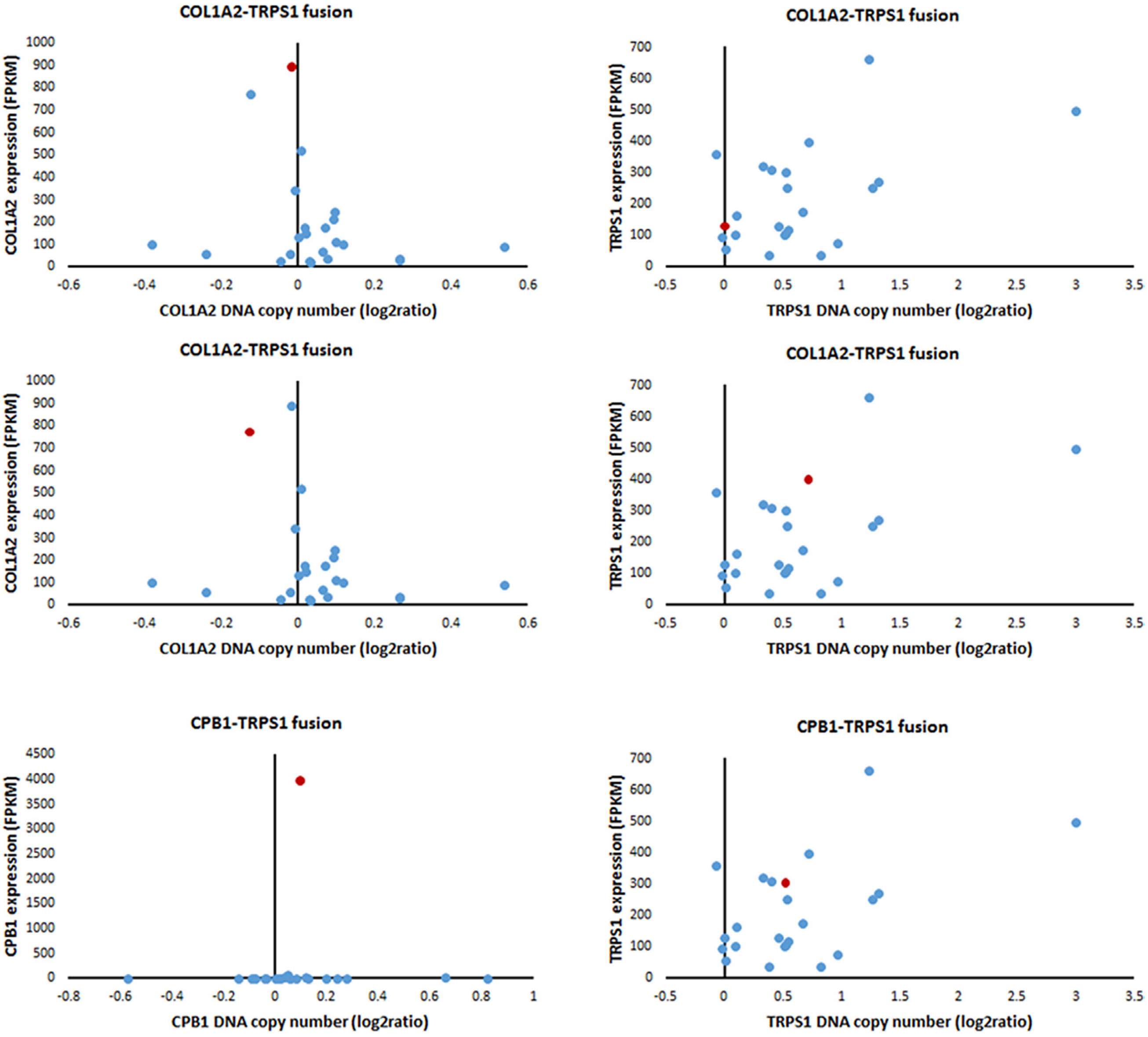
COL1A2-TRPS1 fusion
COL1A2 expression (FPKM)
COL1A2 DNA copy number (log2ratio)
COL1A2-TRPS1 fusion
TRPS1 expression (FPKM)
TRPS1 DNA copy number (log2ratio)
COL1A2-TRPS1 fusion
COL1A2 expression (FPKM)
COL1A2 DNA copy number (log2ratio)
COL1A2-TRPS1 fusion
TRPS1 expression (FPKM)
TRPS1 DNA copy number (log2ratio)
CPB1-TRPS1 fusion
CPB1 expression (FPKM)
CPB1 DNA copy number (log2ratio)
CPB1-TRPS1 fusion
TRPS1 expression (FPKM)
TRPS1 DNA copy number (log2ratio)

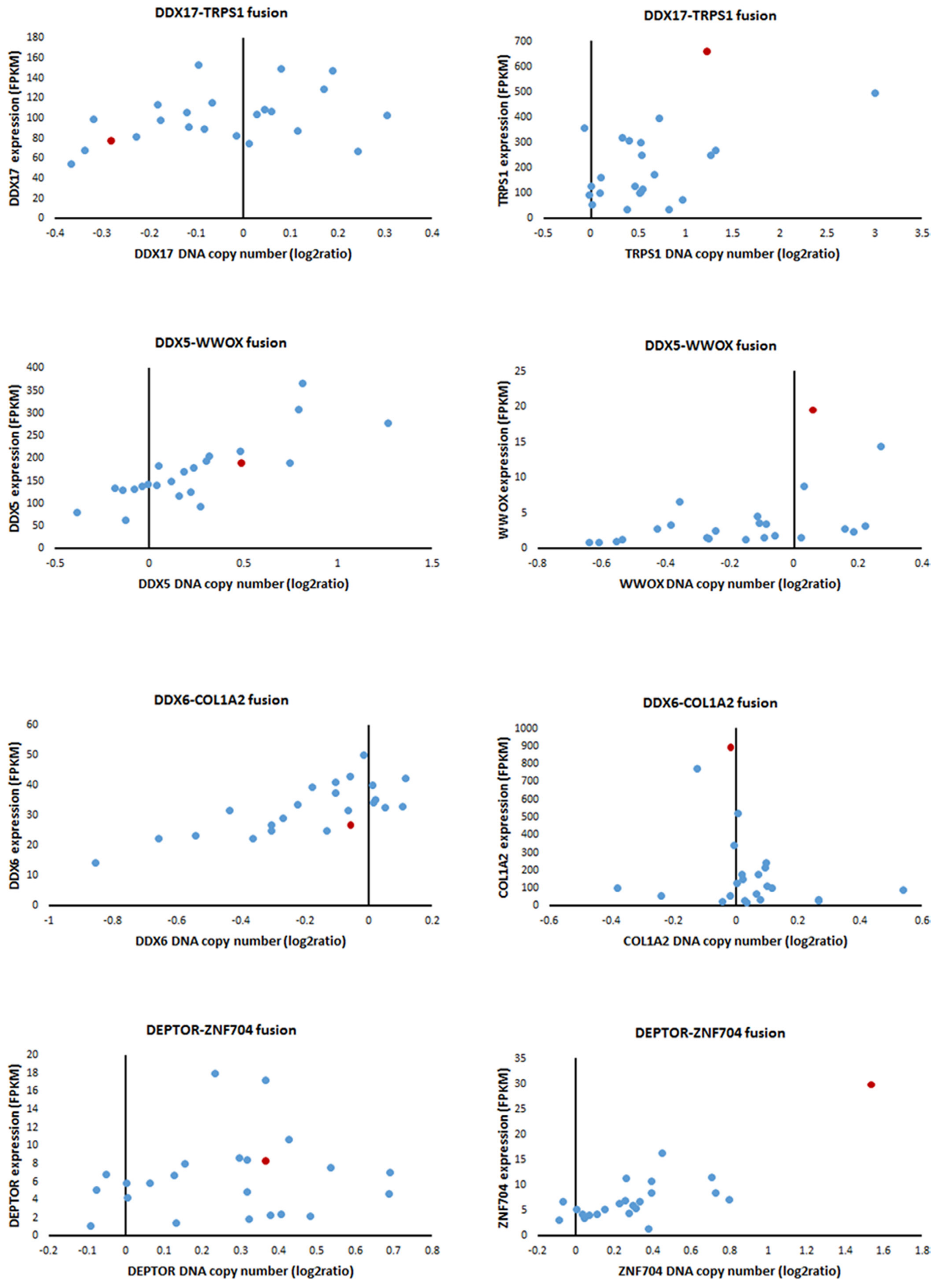
DDX17-TRPS1 fusion
DDX17 expression (FPKM)
DDX17 DNA copy number (log2ratio)
DDX17-TRPS1 fusion
TRPS1 expression (FPKM)
TRPS1 DNA copy number (log2ratio)
DDX5-WWOX fusion
DDX5 expression (FPKM)
DDX5 DNA copy number (log2ratio)
DDX5-WWOX fusion
WWOX expression (FPKM)
WWOX DNA copy number (log2ratio)
DDX6-COL1A2 fusion
DDX6 expression (FPKM)
DDX6 DNA copy number (log2ratio)
DDX6-COL1A2 fusion
COL1A2 expression (FPKM)
COL1A2 DNA copy number (log2ratio)
DEPTOR-ZNF704 fusion
DEPTOR expression (FPKM)
DEPTOR DNA copy number (log2ratio)
DEPTOR-ZNF704 fusion
ZNF704 expression (FPKM)
ZNF704 DNA copy number (log2ratio)

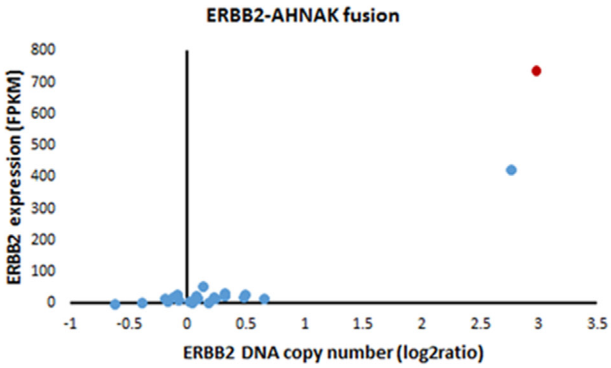
ERBB2-AHNAK fusion
ERBB2 expression (FPKM)
ERBB2 DNA copy number (log2ratio)

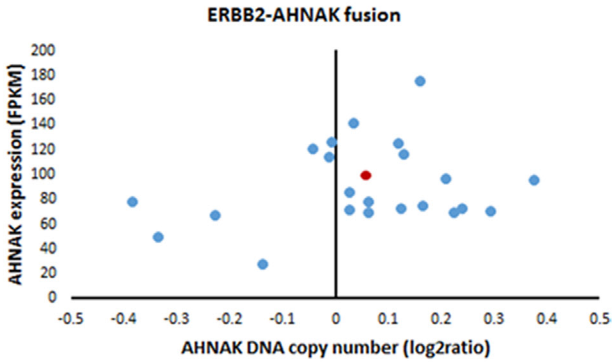
ERBB2-AHNAK fusion
AHNAK expression (FPKM)
AHNAK DNA copy number (log2ratio)

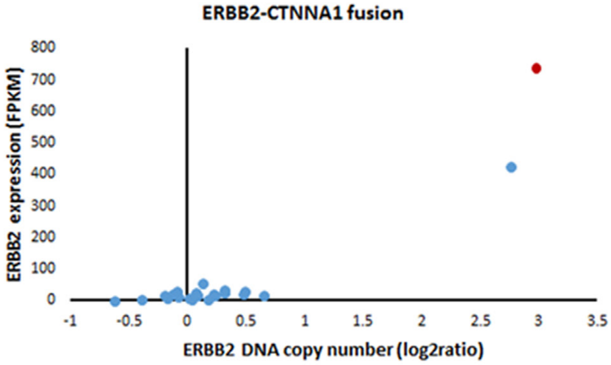
ERBB2-CTNNA1 fusion
ERBB2 expression (FPKM)
ERBB2 DNA copy number (log2ratio)

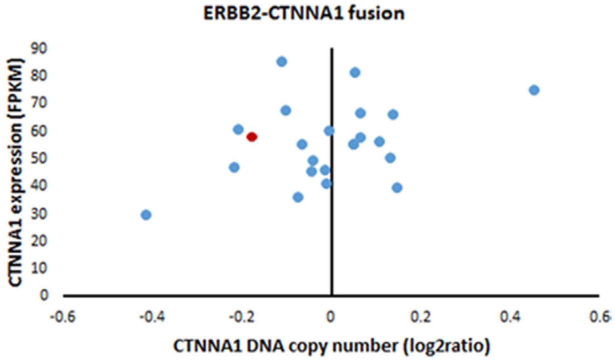
ERBB2-CTNNA1 fusion
CTNNA1 expression (FPKM)
CTNNA1 DNA copy number (log2ratio)

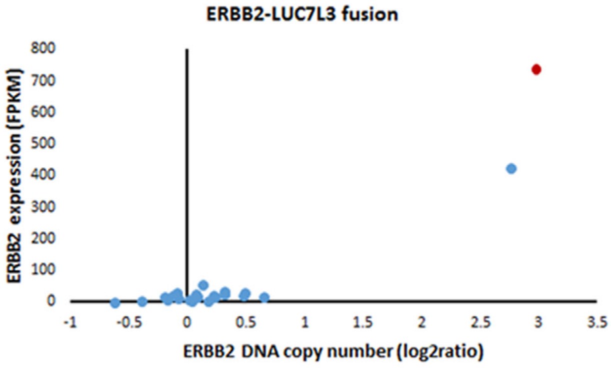
ERBB2-LUC7L3 fusion
ERBB2 expression (FPKM)
ERBB2 DNA copy number (log2ratio)

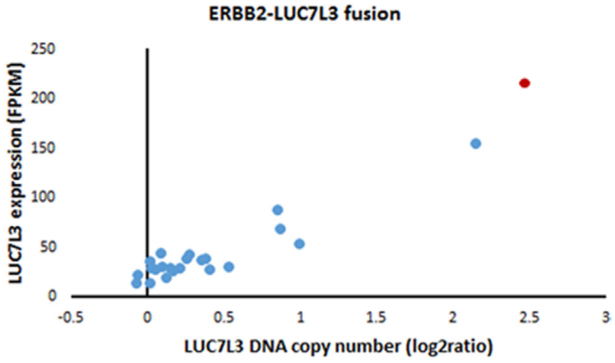
ERBB2-LUC7L3 fusion
LUC7L3 expression (FPKM)
LUC7L3 DNA copy number (log2ratio)

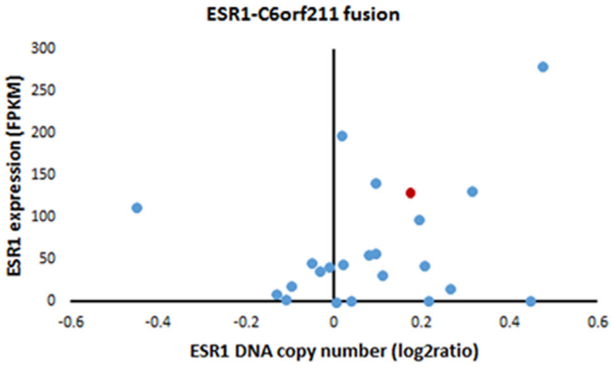
ESR1-C6orf211 fusion
ESR1 expression (FPKM)
ESR1 DNA copy number (log2ratio)

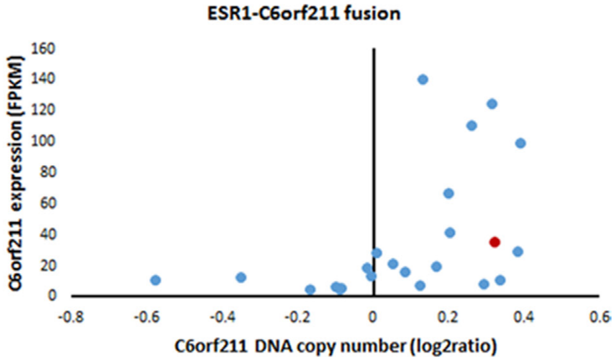
ESR1-C6orf211 fusion
C6orf211 expression (FPKM)
C6orf211 DNA copy number (log2ratio)

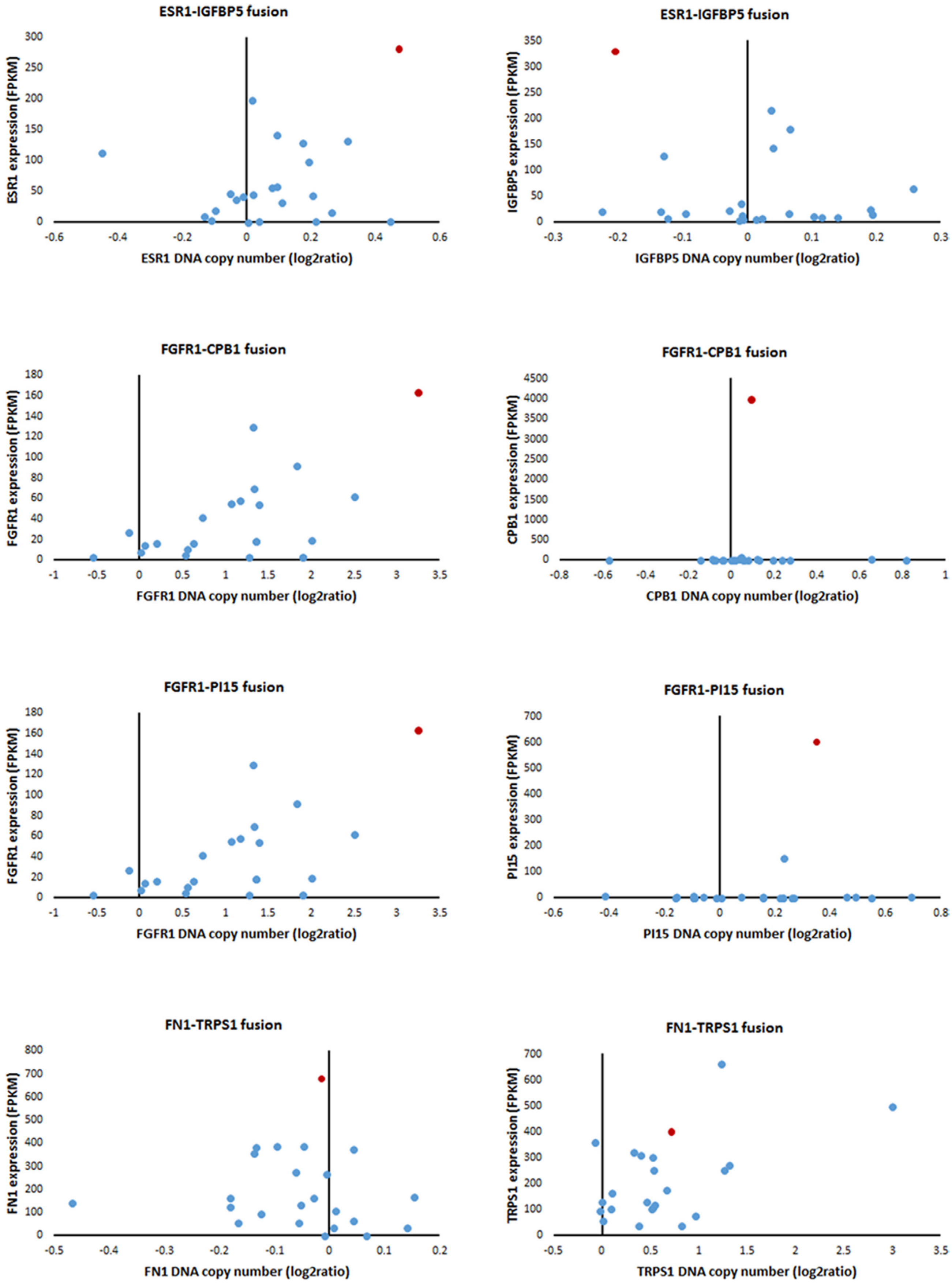
ESR1-IGFBP5 fusion
ESR1 expression (FPKM)
ESR1 DNA copy number (log2ratio)
ESR1-IGFBP5 fusion
IGFBP5 expression (FPKM)
IGFBP5 DNA copy number (log2ratio)
FGFR1-CPB1 fusion
FGFR1 expression (FPKM)
FGFR1 DNA copy number (log2ratio)
FGFR1-CPB1 fusion
CPB1 expression (FPKM)
CPB1 DNA copy number (log2ratio)
FGFR1-PI15 fusion
FGFR1 expression (FPKM)
FGFR1 DNA copy number (log2ratio)
FGFR1-PI15 fusion
PI15 expression (FPKM)
PI15 DNA copy number (log2ratio)
FN1-TRPS1 fusion
FN1 expression (FPKM)
FN1 DNA copy number (log2ratio)
FN1-TRPS1 fusion
TRPS1 expression (FPKM)
TRPS1 DNA copy number (log2ratio)

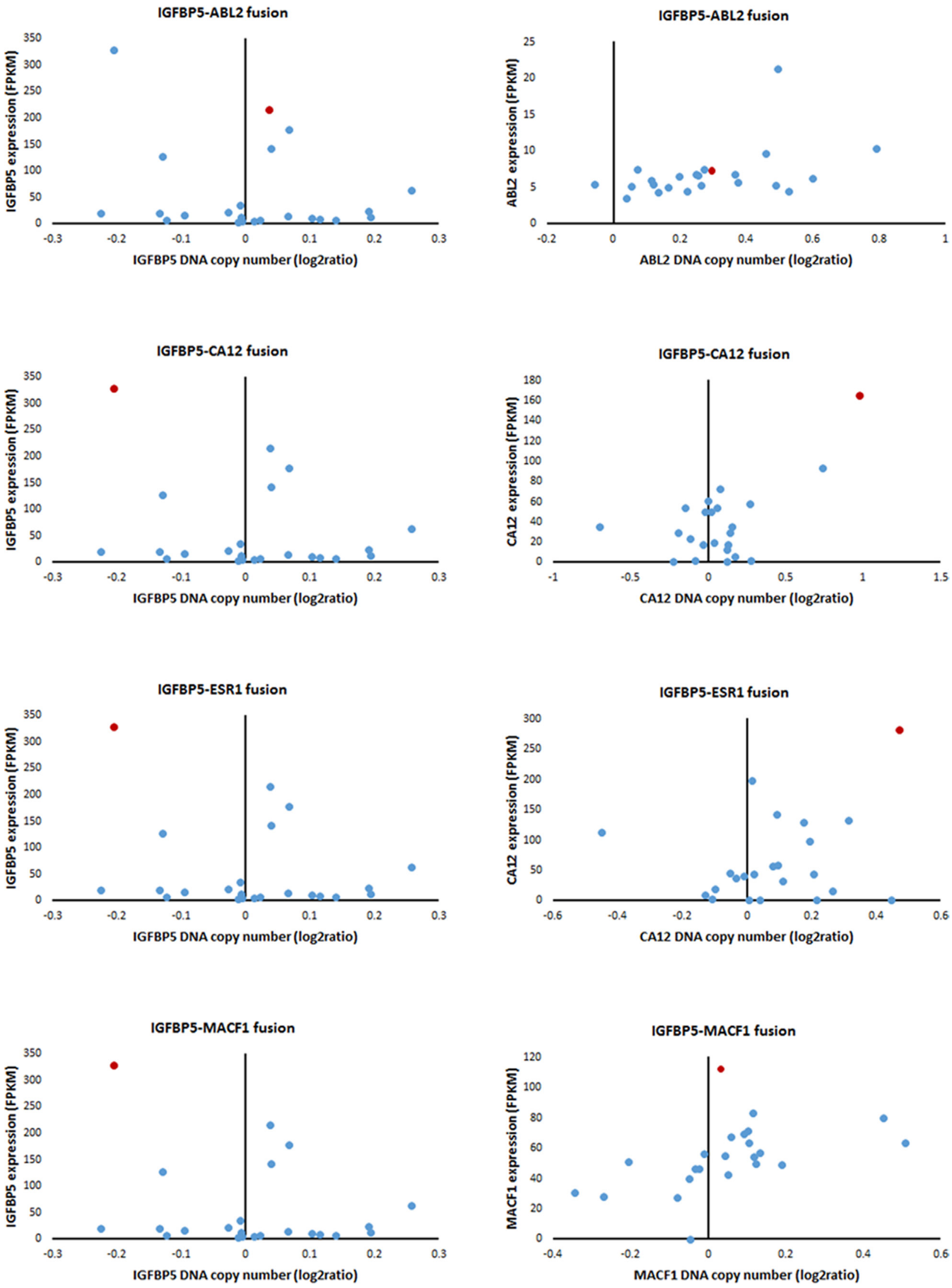
IGFBP5-ABL2 fusion
IGFBP5 expression (FPKM)
IGFBP5 DNA copy number (log2ratio)
IGFBP5-ABL2 fusion
ABL2 expression (FPKM)
ABL2 DNA copy number (log2ratio)
IGFBP5-CA12 fusion
IGFBP5 expression (FPKM)
IGFBP5 DNA copy number (log2ratio)
IGFBP5-CA12 fusion
CA12 expression (FPKM)
CA12 DNA copy number (log2ratio)
IGFBP5-ESR1 fusion
IGFBP5 expression (FPKM)
IGFBP5 DNA copy number (log2ratio)
IGFBP5-ESR1 fusion
CA12 expression (FPKM)
CA12 DNA copy number (log2ratio)
IGFBP5-MACF1 fusion
IGFBP5 expression (FPKM)
IGFBP5 DNA copy number (log2ratio)
IGFBP5-MACF1 fusion
MACF1 expression (FPKM)
MACF1 DNA copy number (log2ratio)

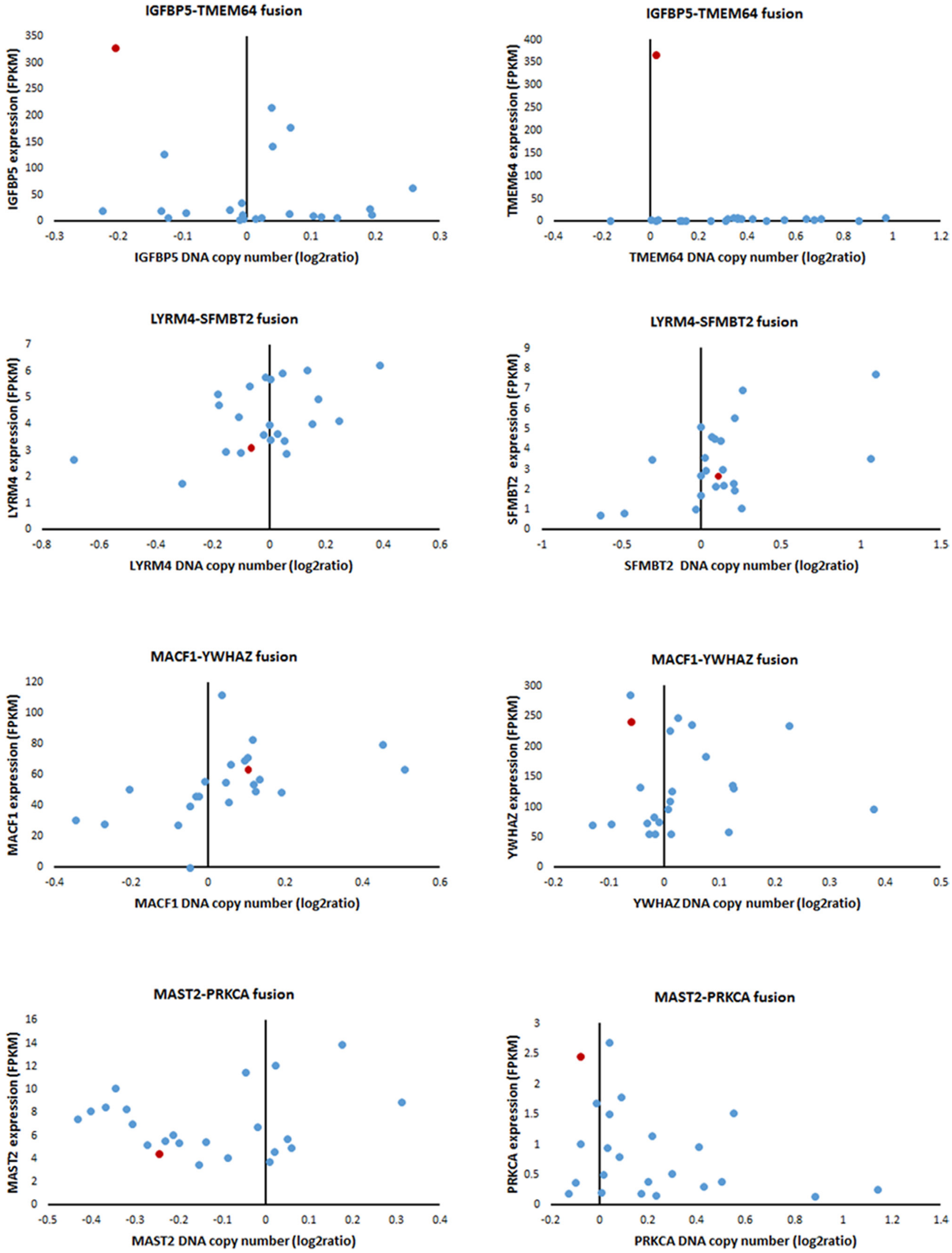
IGFBP5-TMEM64 fusion
IGFBP5 expression (FPKM)
IGFBP5 DNA copy number (log2ratio)
IGFBP5-TMEM64 fusion
TMEM64 expression (FPKM)
TMEM64 DNA copy number (log2ratio)
LYRM4-SFMBT2 fusion
LYRM4 expression (FPKM)
LYRM4 DNA copy number (log2ratio)
LYRM4-SFMBT2 fusion
SFMBT2 expression (FPKM)
SFMBT2 DNA copy number (log2ratio)
MACF1-YWHAZ fusion
MACF1 expression (FPKM)
MACF1 DNA copy number (log2ratio)
MACF1-YWHAZ fusion
YWHAZ expression (FPKM)
YWHAZ DNA copy number (log2ratio)
MAST2-PRKCA fusion
MAST2 expression (FPKM)
MAST2 DNA copy number (log2ratio)
MAST2-PRKCA fusion
PRKCA expression (FPKM)
PRKCA DNA copy number (log2ratio)

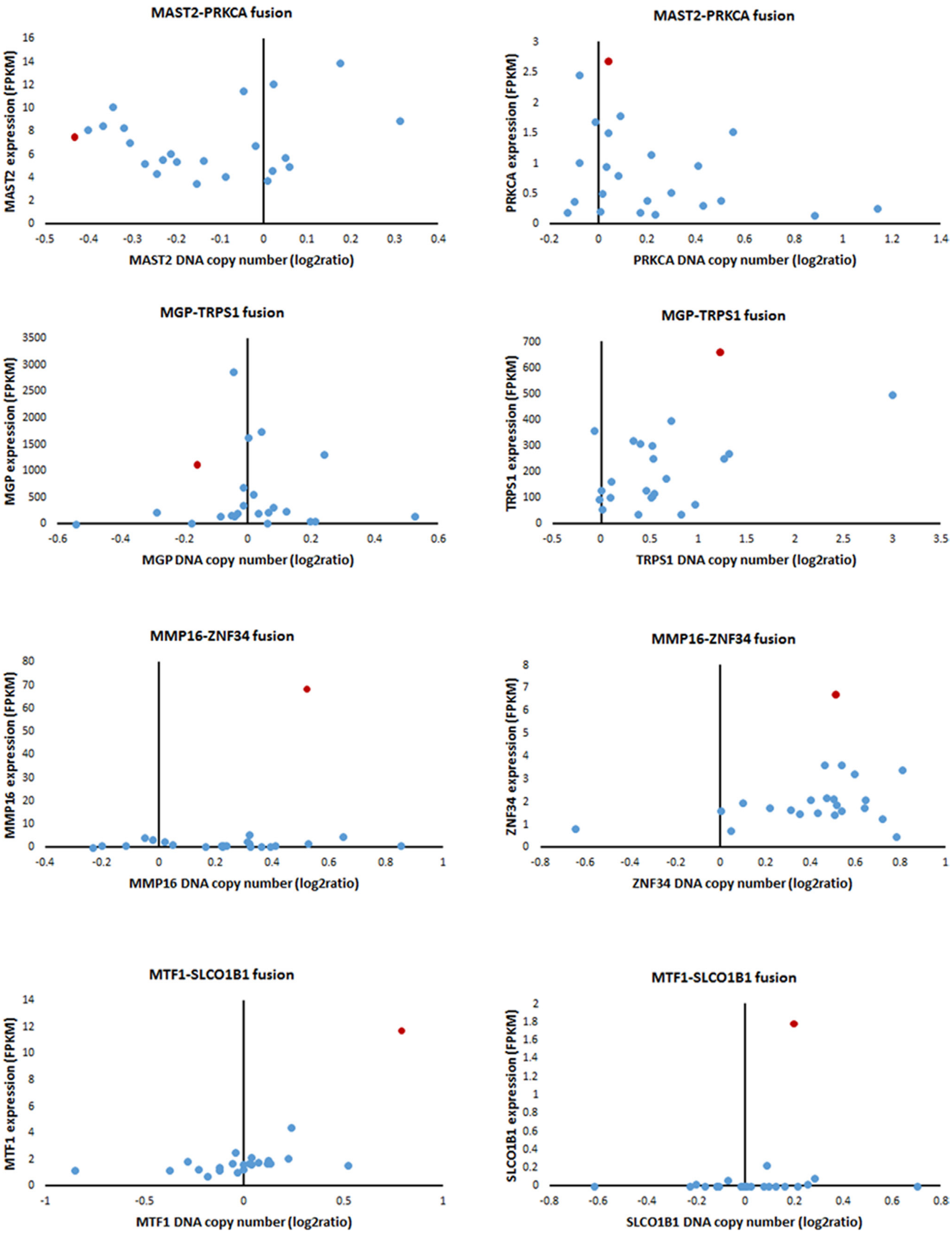
MAST2-PRKCA fusion
MAST2 expression (FPKM)
MAST2 DNA copy number (log2ratio)
MAST2-PRKCA fusion
PRKCA expression (FPKM)
PRKCA DNA copy number (log2ratio)
MGP-TRPS1 fusion
MGP expression (FPKM)
MGP DNA copy number (log2ratio)
MGP-TRPS1 fusion
TRPS1 expression (FPKM)
TRPS1 DNA copy number (log2ratio)
MMP16-ZNF34 fusion
MMP16 expression (FPKM)
MMP16 DNA copy number (log2ratio)
MMP16-ZNF34 fusion
ZNF34 expression (FPKM)
ZNF34 DNA copy number (log2ratio)
MTF1-SLCO1B1 fusion
MTF1 expression (FPKM)
MTF1 DNA copy number (log2ratio)
MTF1-SLCO1B1 fusion
SLCO1B1 expression (FPKM)
SLCO1B1 DNA copy number (log2ratio)

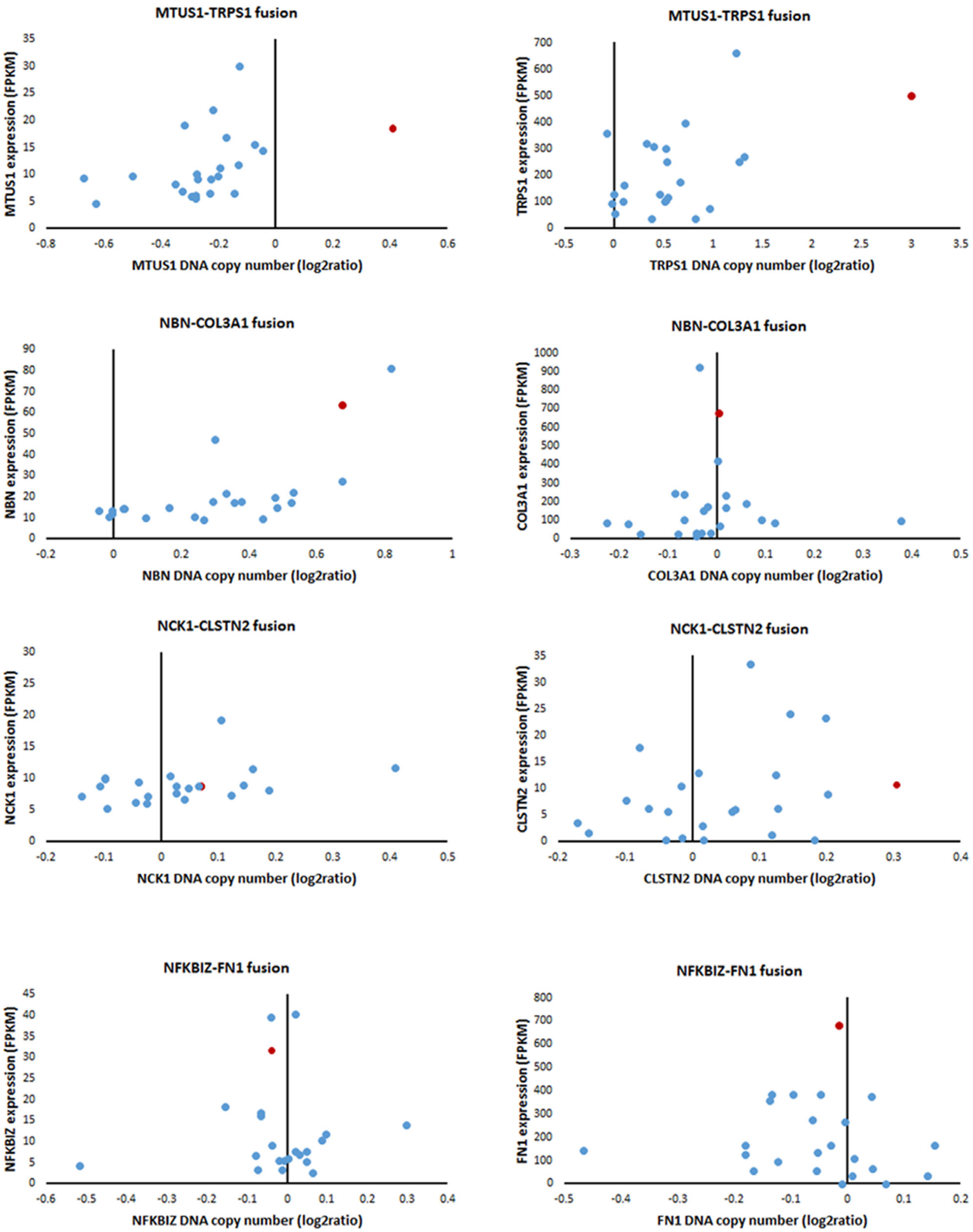
MTUS1-TRPS1 fusion
MTUS1 expression (FPKM)
MTUS1 DNA copy number (log2ratio)
MTUS1-TRPS1 fusion
TRPS1 expression (FPKM)
TRPS1 DNA copy number (log2ratio)
NBN-COL3A1 fusion
NBN expression (FPKM)
NBN DNA copy number (log2ratio)
NBN-COL3A1 fusion
COL3A1 expression (FPKM)
COL3A1 DNA copy number (log2ratio)
NCK1-CLSTN2 fusion
NCK1 expression (FPKM)
NCK1 DNA copy number (log2ratio)
NCK1-CLSTN2 fusion
CLSTN2 expression (FPKM)
CLSTN2 DNA copy number (log2ratio)
NFKBIZ-FN1 fusion
NFKBIZ expression (FPKM)
NFKBIZ DNA copy number (log2ratio)
NFKBIZ-FN1 fusion
FN1 expression (FPKM)
FN1 DNA copy number (log2ratio)

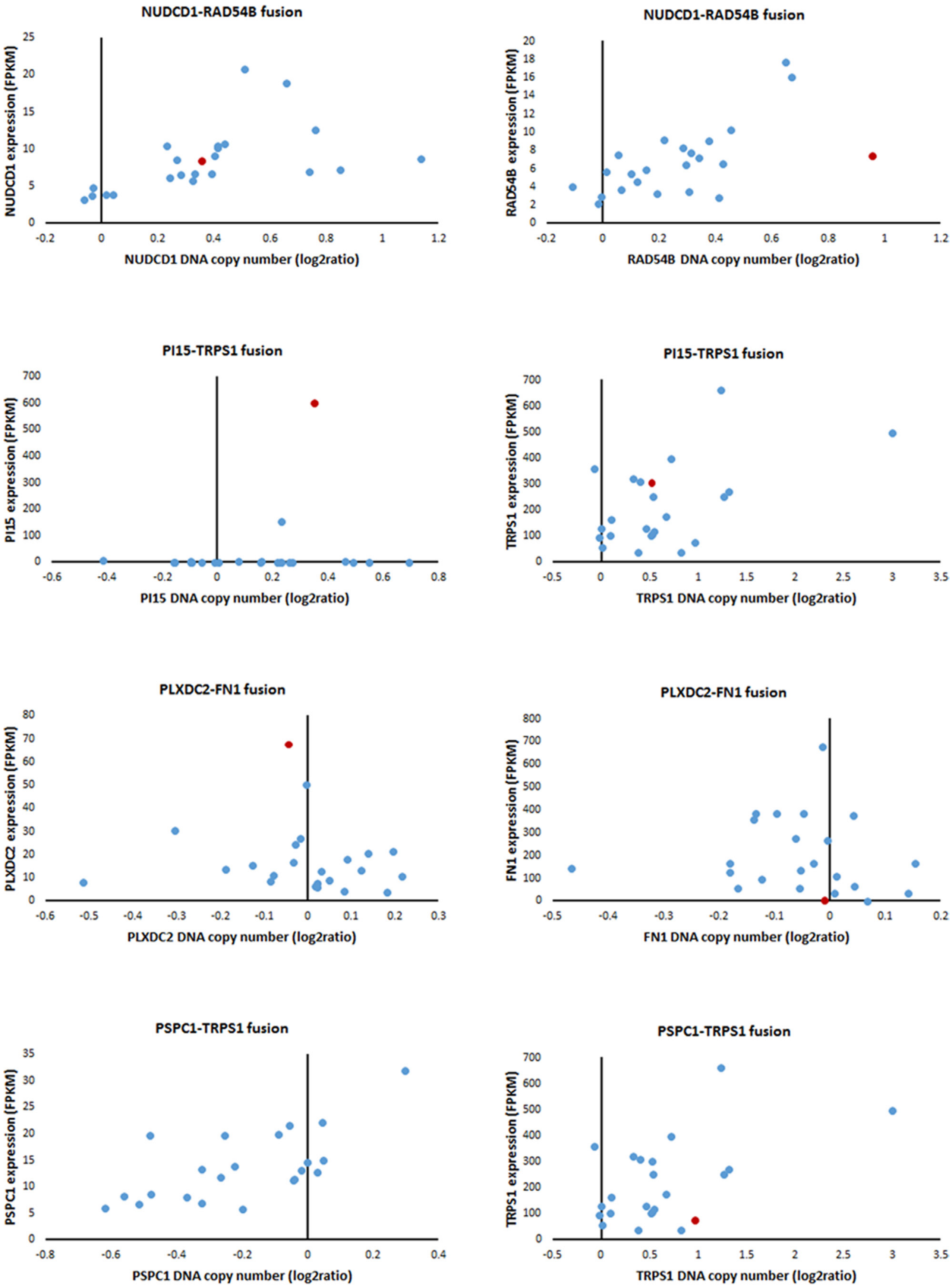
NUDCD1-RAD54B fusion
NUDCD1 expression (FPKM)
NUDCD1 DNA copy number (log2ratio)
NUDCD1-RAD54B fusion
RAD54B expression (FPKM)
RAD54B DNA copy number (log2ratio)
PI15-TRPS1 fusion
PI15 expression (FPKM)
PI15 DNA copy number (log2ratio)
PI15-TRPS1 fusion
TRPS1 expression (FPKM)
TRPS1 DNA copy number (log2ratio)
PLXDC2-FN1 fusion
PLXDC2 expression (FPKM)
PLXDC2 DNA copy number (log2ratio)
PLXDC2-FN1 fusion
FN1 expression (FPKM)
FN1 DNA copy number (log2ratio)
PSPC1-TRPS1 fusion
PSPC1 expression (FPKM)
PSPC1 DNA copy number (log2ratio)
PSPC1-TRPS1 fusion
TRPS1 expression (FPKM)
TRPS1 DNA copy number (log2ratio)

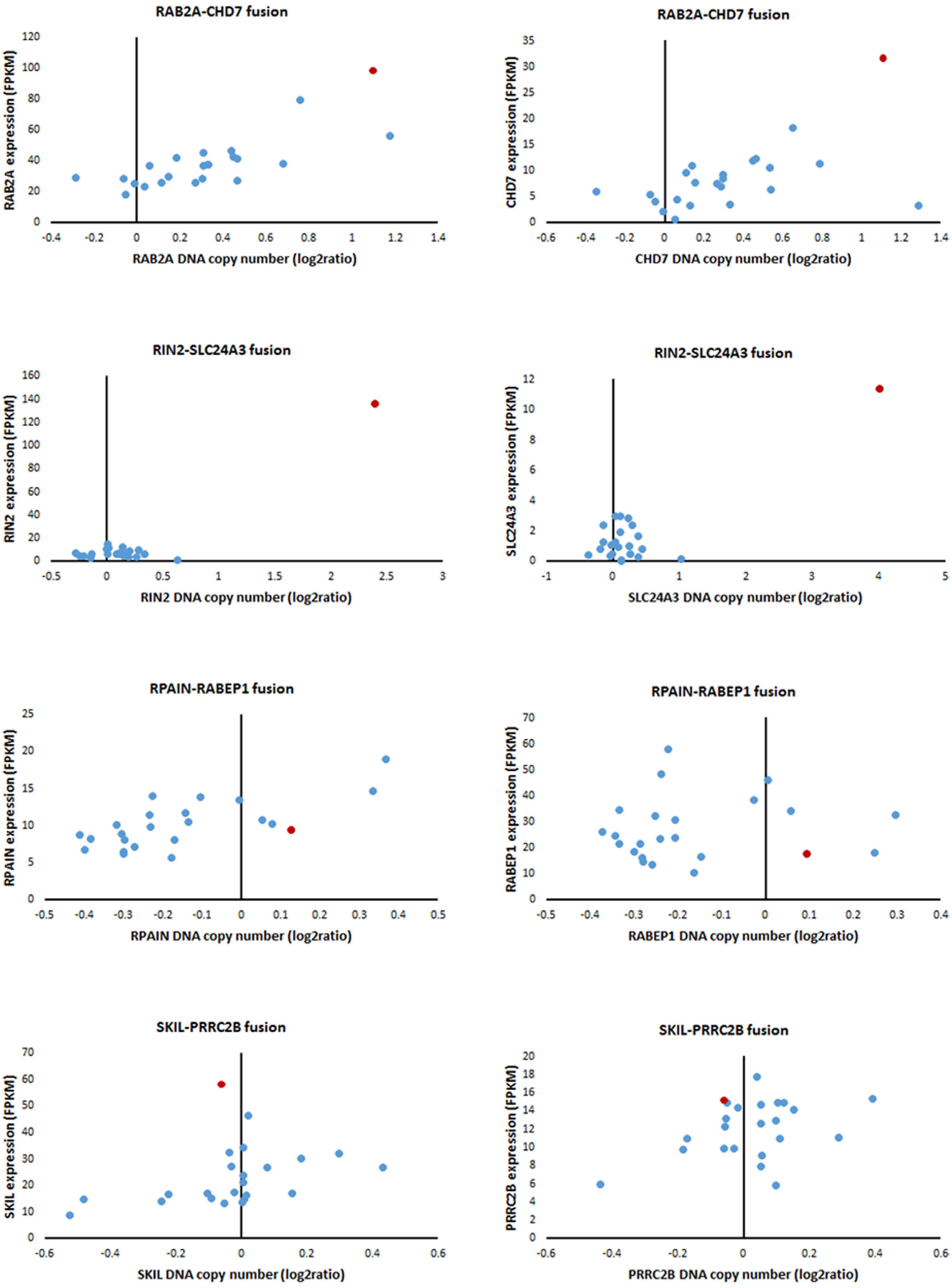
RAB2A-CHD7 fusion
RAB2A expression (FPKM)
RAB2A DNA copy number (log2ratio)
RAB2A-CHD7 fusion
CHD7 expression (FPKM)
CHD7 DNA copy number (log2ratio)
RIN2-SLC24A3 fusion
RIN2 expression (FPKM)
RIN2 DNA copy number (log2ratio)
RIN2-SLC24A3 fusion
SLC24A3 expression (FPKM)
SLC24A3 DNA copy number (log2ratio)
RPAIN-RABEP1 fusion
RPAIN expression (FPKM)
RPAIN DNA copy number (log2ratio)
RPAIN-RABEP1 fusion
RABEP1 expression (FPKM)
RABEP1 DNA copy number (log2ratio)
SKIL-PRRC2B fusion
SKIL expression (FPKM)
SKIL DNA copy number (log2ratio)
SKIL-PRRC2B fusion
PRRC2B expression (FPKM)
PRRC2B DNA copy number (log2ratio)

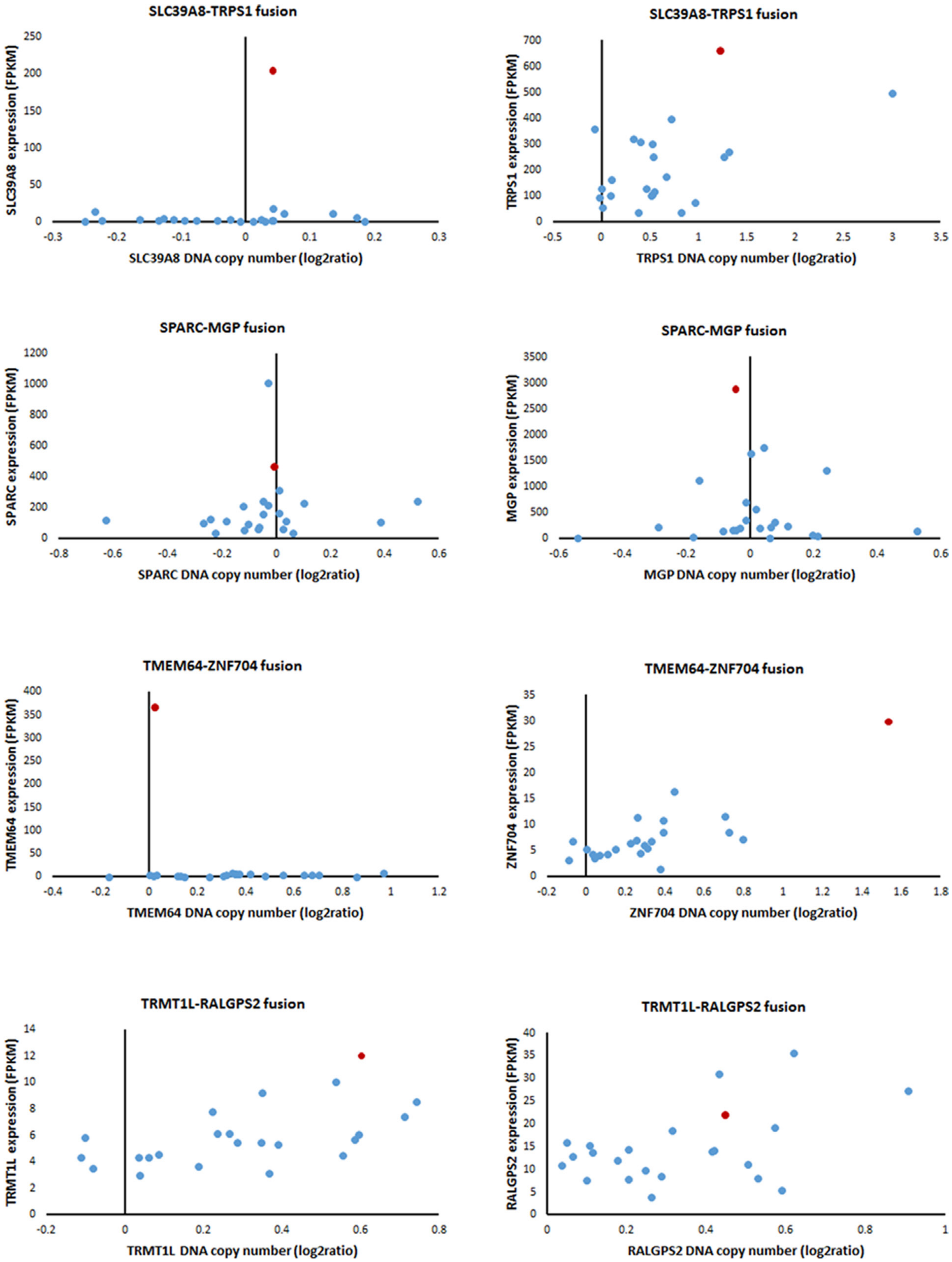
SLC39A8-TRPS1 fusion
SLC39A8 expression (FPKM)
SLC39A8 DNA copy number (log2ratio)
SLC39A8-TRPS1 fusion
TRPS1 expression (FPKM)
TRPS1 DNA copy number (log2ratio)
SPARC-MGP fusion
SPARC expression (FPKM)
SPARC DNA copy number (log2ratio)
SPARC-MGP fusion
MGP expression (FPKM)
MGP DNA copy number (log2ratio)
TMEM64-ZNF704 fusion
TMEM64 expression (FPKM)
TMEM64 DNA copy number (log2ratio)
TMEM64-ZNF704 fusion
ZNF704 expression (FPKM)
ZNF704 DNA copy number (log2ratio)
TRMT1L-RALGPS2 fusion
TRMT1L expression (FPKM)
TRMT1L DNA copy number (log2ratio)
TRMT1L-RALGPS2 fusion
RALGPS2 expression (FPKM)
RALGPS2 DNA copy number (log2ratio)

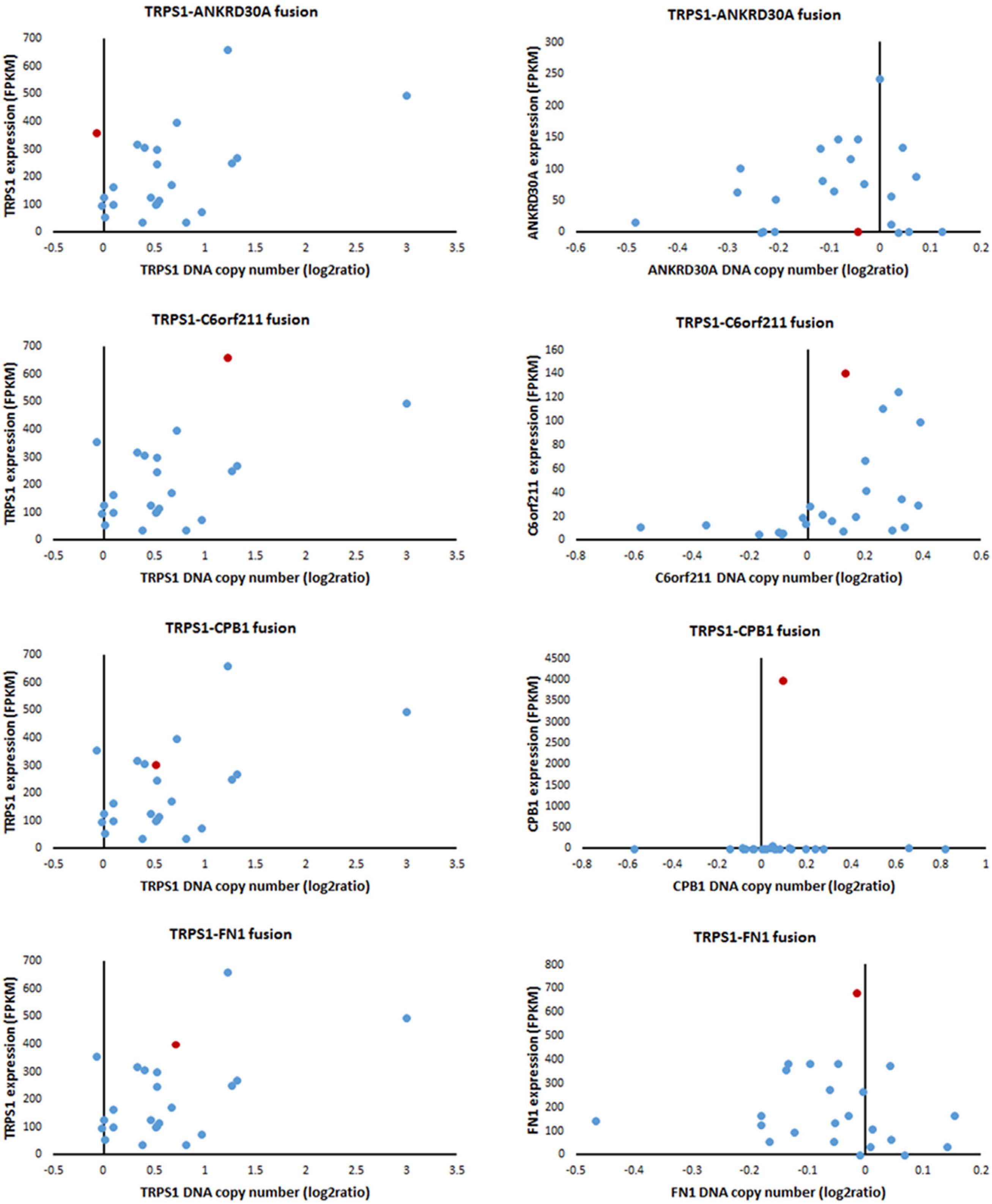
TRPS1-ANKRD30A fusion
TRPS1 expression (FPKM)
0 100 200 300 400 500 600 700
-0.5 0 0.5 1 1.5 2 2.5 3 3.5
TRPS1 DNA copy number (log2ratio)
TRPS1-ANKRD30A fusion
ANKRD30A expression (FPKM)
0 50 100 150 200 250 300
-0.6 -0.5 -0.4 -0.3 -0.2 -0.1 0 0.1 0.2
ANKRD30A DNA copy number (log2ratio)
TRPS1-C6orf211 fusion
TRPS1 expression (FPKM)
0 100 200 300 400 500 600 700
-0.5 0 0.5 1 1.5 2 2.5 3 3.5
TRPS1 DNA copy number (log2ratio)
TRPS1-C6orf211 fusion
C6orf211 expression (FPKM)
0 20 40 60 80 100 120 140 160
-0.8 -0.6 -0.4 -0.2 0 0.2 0.4 0.6
C6orf211 DNA copy number (log2ratio)
TRPS1-CPB1 fusion
TRPS1 expression (FPKM)
0 100 200 300 400 500 600 700
-0.5 0 0.5 1 1.5 2 2.5 3 3.5
TRPS1 DNA copy number (log2ratio)
TRPS1-CPB1 fusion
CPB1 expression (FPKM)
0 500 1000 1500 2000 2500 3000 3500 4000 4500
-0.8 -0.6 -0.4 -0.2 0 0.2 0.4 0.6 0.8 1
CPB1 DNA copy number (log2ratio)
TRPS1-FN1 fusion
TRPS1 expression (FPKM)
0 100 200 300 400 500 600 700
-0.5 0 0.5 1 1.5 2 2.5 3 3.5
TRPS1 DNA copy number (log2ratio)
TRPS1-FN1 fusion
FN1 expression (FPKM)
0 100 200 300 400 500 600 700 800
-0.5 -0.4 -0.3 -0.2 -0.1 0 0.1 0.2
FN1 DNA copy number (log2ratio)

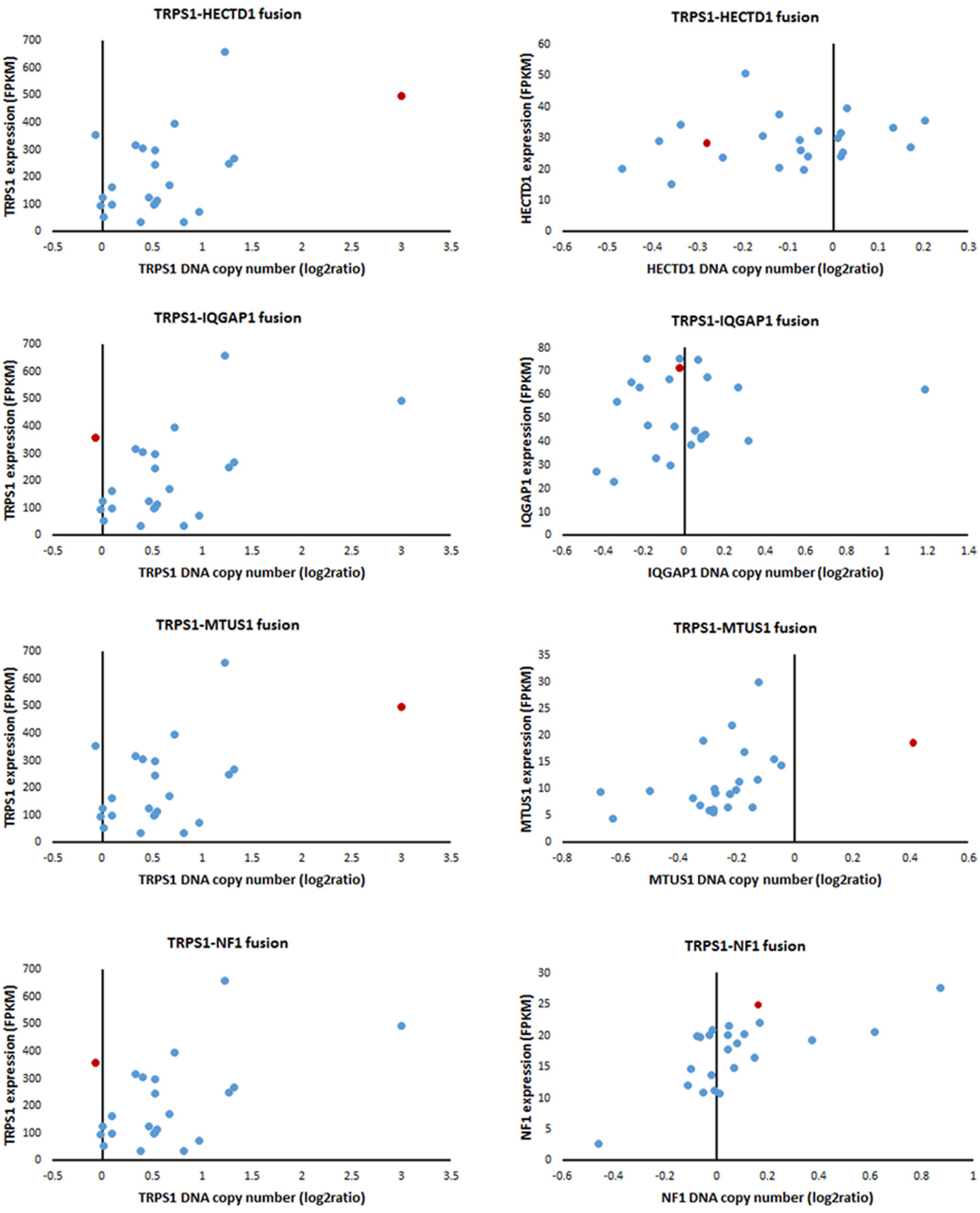
TRPS1-HECTD1 fusion
TRPS1 expression (FPKM)
TRPS1 DNA copy number (log2ratio)
TRPS1-HECTD1 fusion
HECTD1 expression (FPKM)
HECTD1 DNA copy number (log2ratio)
TRPS1-IQGAP1 fusion
TRPS1 expression (FPKM)
TRPS1 DNA copy number (log2ratio)
TRPS1-IQGAP1 fusion
IQGAP1 expression (FPKM)
IQGAP1 DNA copy number (log2ratio)
TRPS1-MTUS1 fusion
TRPS1 expression (FPKM)
TRPS1 DNA copy number (log2ratio)
TRPS1-MTUS1 fusion
MTUS1 expression (FPKM)
MTUS1 DNA copy number (log2ratio)
TRPS1-NF1 fusion
TRPS1 expression (FPKM)
TRPS1 DNA copy number (log2ratio)
TRPS1-NF1 fusion
NF1 expression (FPKM)
NF1 DNA copy number (log2ratio)

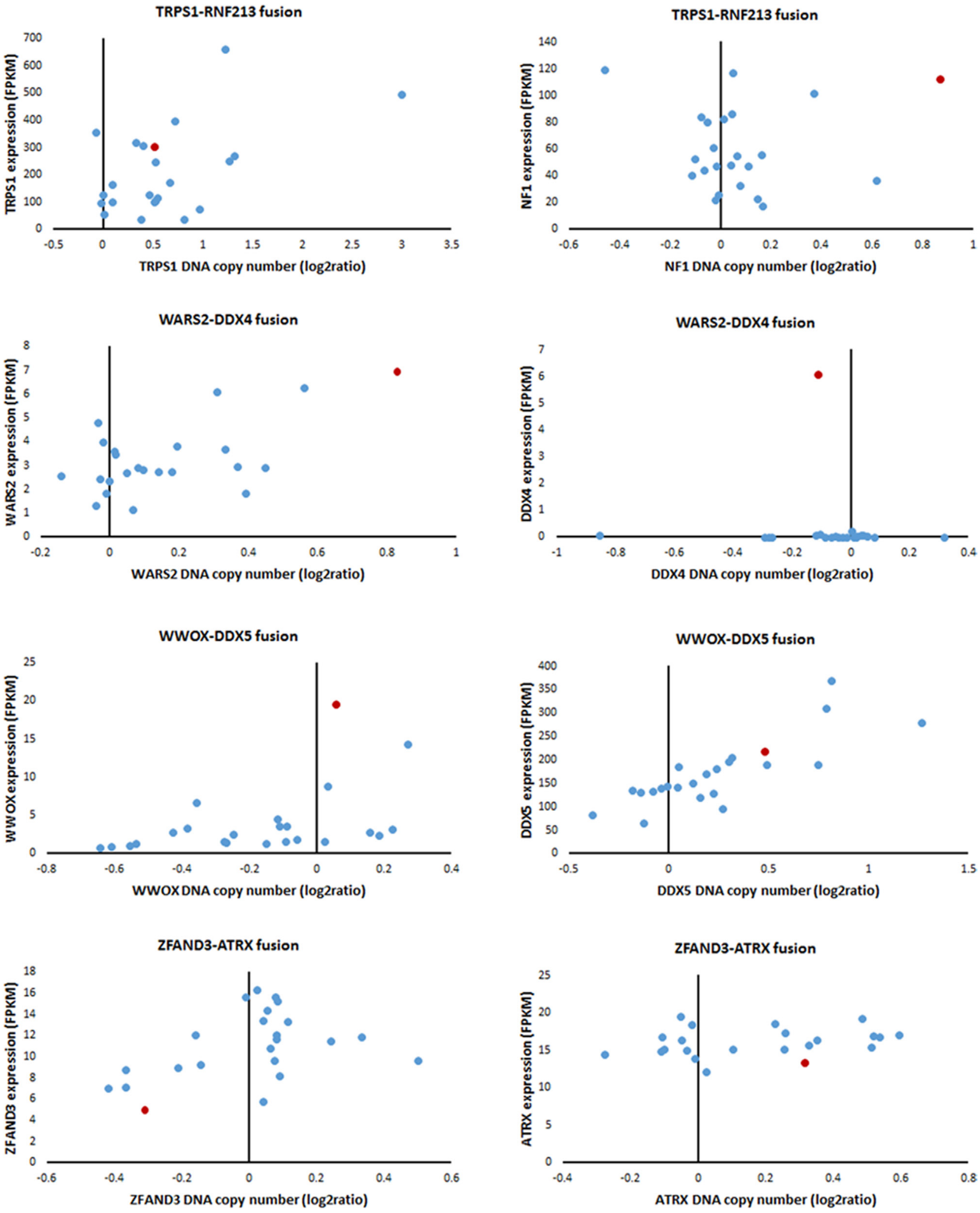

Supplementary Figure 2: Gene expression patterns for gene fusion partners are frequently unrelated to DNA amplification. Scatterplots illustrating gene expression levels (FPKM, y-axis) and DNA copy number (log_2ratio, x-axis) for the 3′- and 5′-gene fusion partners. Red and blue dots depict samples with and without the gene fusion, respectively. DNA amplification is defined as log_2ratio ≥ 0.5.

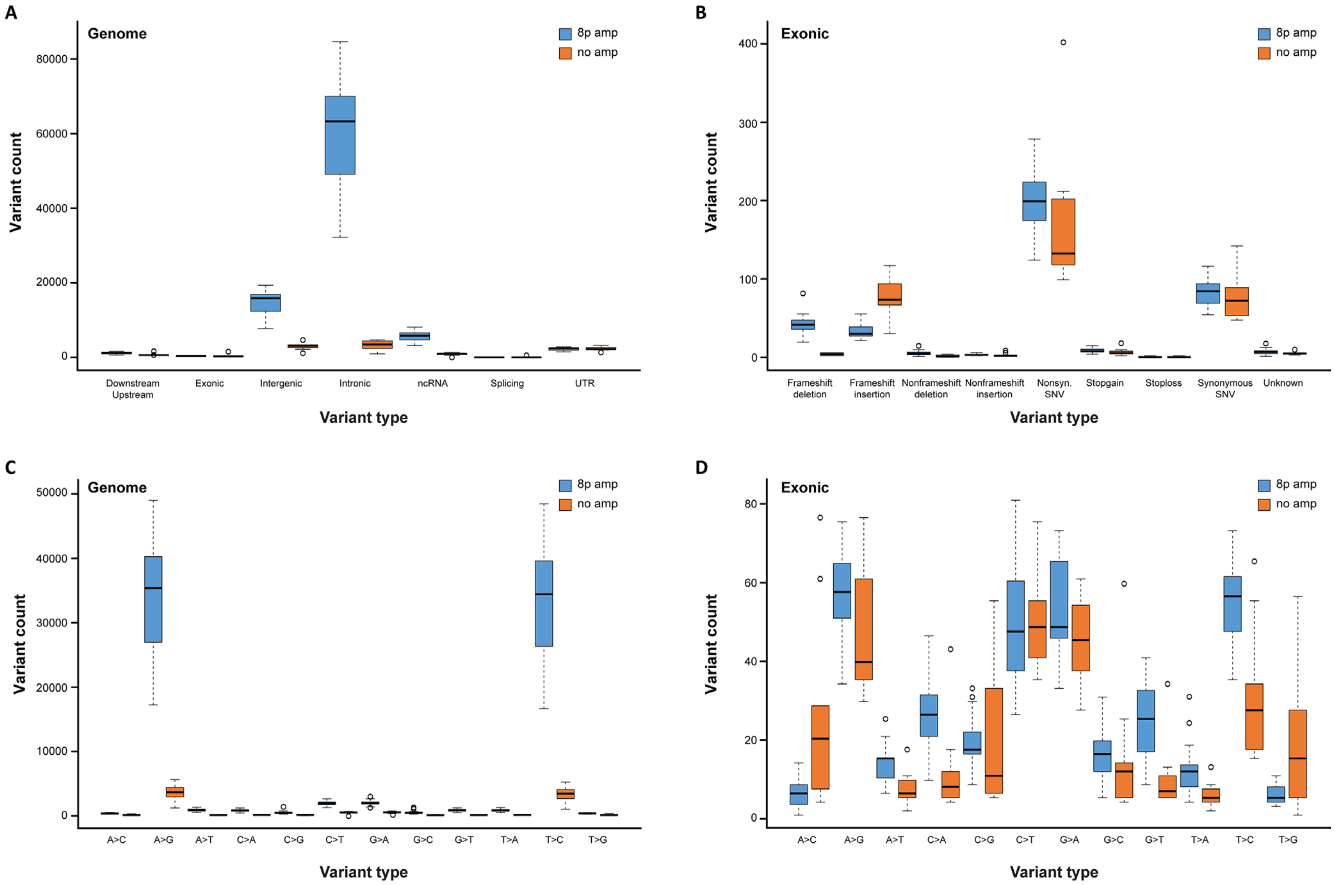

Supplementary Figure 3: Mutational load is independent of 8p11-p12 amplification status in breast carcinomas. Box plots illustrating (**A–B**) the number of genetic variants (y-axis) in genomic and exonic regions (x-axis) for 8p11-p12 amplification (denoted *8p amp*) and the TCGA breast carcinoma samples with neutral 8p11-p12 copy number (denoted *no amp*); and (**C–D**) the number of base-pair substitutions in genomic and exonic regions (y-axis) and the nucleotide change (x-axis).

Supplementary Table 1: Chromothripsis-like events identified in breast carcinomas

Sample	CTLP region	Size (Mb)	Cytoband location	CNA status change times	Likelihood ratio (log10)	Cancer genes
T3010	chr17:30000001-70000000	40.0	17q12-q25.1	29	22	*COL1A1, HLF, CLTC, CD79B, DDX5, PRKAR1A*
T4296	chr11:40000001-128827254	88.8	11p12-q24.3	36	13	*CCND1, PICALM, BIRC3, ATM*
T4672	chr8:30000001-144142980	114.1	8p12-q24.3	49	12	*FGFR1, WHSC1L1, HOOK3, EXT1, MYC, NDRG1*
T5578	chr17:25000001-71944323	46.9	17q11.2-q25.1	20	18	*COL1A1, HLF, MSI2, CLTC, CD79B, DDX5*
T6061	chr8:45000001-145338915	100.3	8p11.1-q24.3	42	34	*NCOA2, HEY1, MYC*
T7008	chr6:95000001-144554710	49.6	6q16.1-q24.2	28	11	*ECT2L, TNFAIP3*
T7008	chr17:38774742-78774742	40.0	17q21.31-q25.3	22	8	*HLF, CLTC, BRIP1, CD79B, PRKAR1A*
T7253	chr8:25000001-65000000	40.0	8p21.2-q12.3	21	13	*FGFR1, WHSC1L1, WRN*
T7253	chr11:50000001-90000000	40.0	11p11.12-q14.3	21	13	*CCND1, NUMA1, PICALM*
T7396	chr17:15000001-77435964	62.4	17p12-q25.3	41	15	*HLF, CLTC, BRIP1, CD79B, DDX5, PRKAR1A, CANT1*
T7396	chr20:30000001-60000000	30.0	20q11.21-q13.33	21	10	*TOP1, GNAS*
T7670	chr6:5000001-119142980	114.1	6p25.1-q22.31	41	9	*DEK, HIST1H41, POU5F1, FANCE, PRDM1*
T7670	chr17:10000001-72435964	62.4	17p13.1-q25.2	28	8	*CDK12, ERBB2, LASP1, MLLT6*
T7678	chr11:30000001-93811651	63.8	11p14.1-q21	31	25	*CCND1, NUMA1, CREB3L1, DDB2, PICALM*
T7682	chr1:46017107-245522847	199.5	1p34.1-q44	41	9	*BCL9, ARNT, MUC1, NTRK1, PRCC, SDHC, MDM4, ELK4, SLC45A3, H3F3A, FH, FUBP1, FAM46C*
T7682	chr11:10000001-124142980	114.1	11p15.4-q24.2	40	17	*CCND1, NUMA1, PICALM, MAML2, ATM, BIRC3, DDX10, POU2AF1, SDHD, ARHGEF12, CBL, DDX6*
T7807	chr6:60000001-136117153	76.1	6p11.1-q23.3	40	14	
T8009	chr8:85000001-131944323	46.9	8q21.2-q24.22	30	12	*COX6C, MYC*
T8009	chr11:55677642-134452384	78.8	11q11-q25	33	9	*CCND1, NUMA1, PICALM, MAML2, ATM, BIRC3, DDX10, POU2AF1, SDHD, ARHGEF12, CBL, DDX6, FLI1*
T8038	chr11:45000001-133827254	88.8	11p11.2-q25	20	20	*CCND1*
T8041	chr17:15000001-77435964	62.4	17p12-q25.3	38	28	*SUZ12, CDK12, ERBB2, HLF, MSI2, CLTC, BRIP1, PRKAR1A, BRCA1, ETV4, RARA, CANT1*
T8277	chr1:1-243018229	243.0	1p36.33-q44	48	36	*MUTYH, JUN, JAK1, FUBP1, NOTCH2, PDE4DIP, BCL9, ARNT, MUC1, NTRK1, PRCC, SDHC, TPR, MDM4, ELK4, SLC45A3*
T8359	chr10:40000001-102435964	62.4	10p11.1-q24.31	22	22	*CCDC6*
T8376	chr11:20000001-126368585	106.4	11p15.1-q24.2	33	19	*CCND1, PAFAH1B2, PCSK7, PICALM, BIRC3, MAML2, ATM, DDX10, POU2AF1, SDHD, ARHGEF12, CBL, DDX6*
T8456	chr11:30000001-130338915	100.3	11p14.1-q25	30	16	*CCND1, EXT2, PICALM, MAML2, ATM, DDX10, POU2AF1, SDHD*
T8491	chr17:15000001-77435964	62.4	17p12-q25.3	27	29	*CDK12, ERBB2, LASP1, MLLT6, ETV4, MSI2, CLTC, BRIP1, CD89B, PRKAR1A*
T8804	chr6:1-170975699	171.0	6p25.3-q27	49	10	
T8828	chr6:75000001-163827254	88.8	6q13-q26	41	9	*PRDM1, GOPC, ROS1, ECT2L, MYB, TNFAIP3, EZR*
T8842	chr1:139154262-245522847	106.4	1q12-q44	20	19	*MDM4, ELK4, SLC45A3, FH*

T8860	chr11:2002573-134452384	132.5	11p15.5-q25	36	10	*CCND1, NUMA1, PICALM, MAML2, ATM, BIRC3, DDX10, POU2AF1, SDHD, PAFAH1B2, PCSK7, ARHGEF12, CBL, DDX6, FLI1*
T8931	chr4:125000001-174554710	49.6	4q28.1-34.1	29	18	*FBXW7*
T8931	chr11:15000001-61944323	46.9	11p15.2-q12.3	21	10	*FANCF*
T8977	chr6:85000001-148811651	63.8	6q14.3-q24.3	36	26	*PRDM1*
T9042	chr11:30000001-93811651	63.8	11p14.1-q21	22	21	*CCND1, PICALM*
T9086	chr7:1-100338915	100.3	7p22.3-q22.1	34	31	*CARD11, PMS2, ETV1, HNRNPA2B1, HOXA11, HOXA13, HOXA9, AKAP9, CDK6*
T9494	chr17:38774742-78774742	40.0	17q21.31-q25.3	22	8	*MSI2, CLTC, BRIP1, CD79B, DDX5, PRKAR1A, ASPSCR1*
T9612	chr11:25000001-125338915	100.3	11p14.3-q24.2	20	9	*CCND1, PAFAH1B2, PCSK7*
T9632	chr1:145000001-245338915	100.3	1q21.1-q44	34	10	*ARNT, TPM3, PRCC, SDHC, PBX1, ABL2, TPR, ELK4, MDM4, SLC45A3, H3F3A, FH*
T9769	chr11:65000001-95000000	30.0	11q13.1-q21	20	12	*CCND1*
T9806	chr11:15000001-91117153	76.1	11p15.2-q14.3	21	21	*EXT2, PICALM*
T10400	chr11:10000001-124142980	114.1	11p15.4-q24.2	44	14	*CCND1, PAFAH1B2, PCSK7, WT1, NUMA1, PICALM, MAML2, BIRC3, ATM, DDX10, POU2AF1, SDHD, DDX6, CBL, ARHGEF12*
T10457	chr11:25000001-131368585	106.4	11p14.3-q25	21	22	*CCND1, NUMA1*
T10737	chr12:20000001-82435964	62.4	12p12.2-q21.31	21	20	*MDM2*
T10823	chr8:7845558-146274826	138.4	8p23.1-q24.3	73	14	*PCM1, HOOK3, TCEA1, CHCHD7, PLAG1, NCOA2, EXT1, MYC, WRN, WHSC1L1, NDRG1*
T10823	chr17:15000001-77435964	62.4	17p12-q25.3	46	13	*MSI2, CLTC, BRIP1, CD79B, DDX5, PRKAR1A, CANT1, NF1, RAF15, CDK12, ERBB2, RARA*
T10900	chr19:1375687-63811651	62.4	19p13.3-q13.43	40	8	*MLLT1, SH3GL1, DNM2, SMARCA4, BRD4, TPM4, JAK3, ELL, CEBPA, AKT2, BCL3, CBLC, ERCC2, KLK2, TFPT*
T10904	chr11:35000001-97435964	62.4	11p13-q22.1	31	35	*MEN1, CCND1, PICALM*
T11239	chr2:1-243018229	243.0	2p25.3-q37.3	95	28	*ACSL3*
T11248	chr11:40000001-128827254	88.8	11p12-q24.3	25	14	*CCND1, NUMA1, PICALM, ATM, BIRC3, DDX10, MAML2, POU2AF1, SDHD, ARHGEF12, CBL, DDX6, FLI1*
T11292	chr1:105000001-243429268	138.4	1p21.1-q44	39	8	*FAM46C, NOTCH2, TPE4DIP, BCL9, ARNT, NTRK1, PRCC, SDHC, PBX1, ABL2, TPR, MDM4, ELK4, SLC45A3, H3F3A, FH*
T11314	chr8:60000001-136117153	76.1	8q12.1-q24.22	37	9	*EXT1, MYC*
T11315	chr8:75000001-137435964	62.4	8q21.11-q24.23	28	9	*HEY1, EXT1, MYC, NDRG1*
T11378	chr17:2657589-78774742	76.1	17p13.3-q25.3	33	11	*SUZ12, CDK12, ERBB2, COL1A1, HLF, MSI2, BRIP1, CLTC, CD79B, DDX5, PRKAR1A, CANT1*
T11633	chr11:55000001-85000000	30.0	11q11-q14.1	22	11	CCND1
T11659	chr8:30000001-144142980	114.1	8p12-q24.3	44	8	FGFR1, WHSC1L1, HOOK3, TCEA1, CHCHD7, PLAG1, NCOA2, HEY1, COX6C, EXT1, MYC, NDRG1, WRN
T11742	chr8:85000001-131944323	46.9	8q21.2-q24.22	27	28	*MYC*
T11742	chr11:2002573-134452384	132.5	11p15.5-q25	39	22	*LMO2, FLI1*
T11881	chr11:20000001-126368585	106.4	11p15.1-q24.2	29	19	*CCND1, PAFAH1B2, PCSK7*

Supplementary Table 2: Recurrent regions of amplification within the 8p11-p12 amplicon

Current study (*n* = 47)								
**Amplicon Peak**	**Region**	**Size (kb)**	**Cytoband Location**	**Genes mapped to region**	**Number of genes**	**Frequency %**	**% of CNV overlap**	***P*-value**
P1	chr8:34334337-34443082	108.7	8p12		0	30	0	0
P2	chr8:37351420-37571832	220.4	8p12		0	72	16	0
P3	chr8:37676135-37899290	223.2	8p12	*ERLIN2, LOC728024, PROSC, GPR124, BRF2, RAB11FIP1*	6	72	2	0
P4	chr8:38260728-38328670	67.9	8p12	*WHSC1L1*	1	74	0	0
P5	chr8:38498119-38729433	231.3	8p12 - p11.23	*C8orf86, RNF5P1, TACC1*	3	72	64	0
P6	chr8:40184216-40278522	94.3	8p11.21		0	53	5	0
P7	chr8:40780622-40960961	180.3	8p11.21	*ZMAT4*	1	49	0	0
P8	chr8:41752765-41793948	41.2	8p11.21	*ANK1*	1	47	0	0
P9	chr8:42099873-42477259	377.4	8p11.21	*AP3M2, PLAT, IK8K8, POLB, DKK4, VDAC3, SLC20A2*	7	43	8	0

Supplementary Table 3: Bacterial artificial chromosomes (BACs) used in the FISH experiments

BAC clone	Gene(s) name	Start (bp)	End (bp)	Size (bp)	Band
RP11-380B11	*FKSG2;KCNU1*	36836720	37003025	166306	8p12
RP11-745K6	*KCNU1*	36911752	37077533	165782	8p12
RP11-371M15	*NULL*	36980612	37176230	195619	8p12
RP11-95I18	*NULL*	37153188	37307820	154633	8p12
RP11-527N22	*NULL*	37193796	37395020	201225	8p12
RP11-621B1	*NULL*	37365132	37518160	153029	8p12
RP13-631H19	*NULL*	37399410	37535863	136454	8p12
RP11-332C8	*LOC157860*	37418780	37607801	189022	8p12
RP11-319J12	*LOC157860;FLJ14299*	37481145	37696334	215190	8p12
RP13-509O17	*LOC157860*	37517260	37655936	138677	8p12
RP13-620O23	*LOC157860;FLJ14299*	37560498	37710298	149801	8p12
CTD-2015B18	*SPFH2;FLJ14299*	37626978	37730745	103768	8p12
RP13-580P15	*SPFH2;PROSC;GPR124*	37718514	37794138	75625	8p12
RP11-168H8	*BRF2;SPFH2;PROSC;GPR124;RAB11FIP1*	37720525	37874916	154392	8p12
CTD-2225N15	*MGC33309;BRF2;GPR124;RAB11FIP1*	37790755	37923664	132910	8p12
RP11-156L3	*MGC33309;EIF4EBP1;ADRB3;RAB11FIP1*	37852536	38058325	205790	8p12
RP11-594D10	*EIF4EBP1;ASH2L;ADRB3*	37934266	38102965	168700	8p12
RP11-389E22	*EIF4EBP1;STAR;ASH2L;BAG4;LSM1*	38035380	38189049	153670	8p12
RP11-601G22	*DDHD2;WHSC1L1;BAG4;LSM1;HTPAP*	38128912	38295034	166123	8p12
RP11-636F12	*DDHD2;WHSC1L1;BAG4;HTPAP*	38168966	38329500	160535	8p12
CTD-2385A20	*DDHD2;WHSC1L1;HTPAP*	38209555	38298495	88941	8p12
RP11-350N15	*LETM2;WHSC1L1;FLJ43582;FGFR1*	38289075	38488995	199921	8p12
RP11-148D21	*LETM2;WHSC1L1;FLJ43582;FGFR1*	38307871	38507244	199374	8p12 - 8p11.23
RP11-359P11	*FLJ43582;FGFR1*	38427815	38603011	175197	8p12 - 8p11.23
RP11-675F6	*FLJ43582*	38463692	38632874	169183	8p12 - 8p11.23
RP11-734M8	*NULL*	38529206	38697350	168145	8p11.23
RP11-495010	*NULL*	38549335	38761516	212182	8p11.23
RP11-794F5	*TACC1*	38593558	38776969	183412	8p11.23
RP11-690P9	*TACC1*	38640388	38776969	136582	8p11.23
RP11-732D22	*TACC1;PLEKHA2*	38725911	38893939	168029	8p11.23
CTD-2544N14	*ADAM9;HTRA4;PLEKHA2;BLP1*	38871774	39070960	199187	8p11.23
RP11-742F8	*ADAM9;HTRA4;PLEKHA2;BLP1;ADAM32*	38922348	39109975	187628	8p11.23
RP11-391D11	*ADAM9;HTRA4;BLP1;ADAM32*	38954288	39152222	197935	8p11.23
RP11-742H5	*ADAM9;ADAM32*	39053922	39235325	181404	8p11.23
CTD-2172C13	*ADAM5;ADAM32*	39186741	39349390	162650	8p11.23
RP11-583G7	*ADAM5;ADAM32*	39221703	39398472	176770	8p11.23
RP11-33N21	*FLJ30656;ADAM3A*	39420279	39553850	133572	8p11.23 - 8p11.22
RP11-1152G18	*ADAM18*	39510564	39647698	137135	8p11.22
RP11-769N8	*ADAM18*	39541437	39717022	175586	8p11.22
RP11-676B15	*NULL*	39569848	39628893	59046	8p11.22
RP11-648J12	*ADAM2;ADAM18*	39587562	39768413	180852	8p11.22
RP11-143C18	*ADAM2;ADAM18*	39644486	39797061	152576	8p11.22
RP11-44K6	*LOC169355;INDO*	39836599	39985605	149007	8p11.22 - 8p11.21
RP11-157N22	*NULL*	39810942	40004703	193762	8p11.22 - 8p11.21
RP11-719N14	*C8orf4;LOC169355*	39973683	40163169	189487	8p11.21
RP11-508J20	*C8orf4*	40056046	40205264	149219	8p11.21

CTD-2170K14	*NULL*	40146465	40212249	65785	8p11.21
RP11-104D16	*NULL*	40154516	40344796	190281	8p11.21
RP11-812C6	*NULL*	40275993	40464496	188504	8p11.21
RP11-108H10	*FLJ13842*	40395268	40567036	171769	8p11.21
RP11-51K12	*FLJ13842*	40501065	40649254	148190	8p11.21
RP11-592B24	*NULL*	40583114	40764962	181849	8p11.21
RP11-749P1	*NULL*	40620821	40796283	175463	8p11.21
RP11-284J3	*NULL*	40666764	40830435	163672	8p11.21
RP11-465K16	*FLJ13842*	40778859	40972561	193703	8p11.21
RP11-167P2	*FLJ13842*	40787605	40949362	161758	8p11.21
RP11-681J6	*NULL*	40916540	41110713	194174	8p11.21
CTD-2035O24	*SFRP1*	41110715	41238819	128105	8p11.21
RP11-642F7	*MALAT1*	65266462	65429053	162591	11q13.1
RP11-1104L6	*MALAT1*	65200229	65378762	178533	11q13.1
RP11-472D15	*MALAT1*	65264768	65455761	190993	11q13.1
CTD-2240J20	*AHNAK*	62191334	62336912	145578	11q12.3
RP11-466M24	*TRPS1*	116341665	116522802	181137	8q23.3
RP11-762A3	*TRPS1*	116485587	116668671	183084	8q23.3
RP11-794B13	*FOXP1*	71180816	71356061	175245	3p13
RP11-905F6	*FOXP1*	71256411	71475547	219136	3p13
RP11-109G20	*FOXP1*	71027347	71179919	152572	3p13
RP11-1127D11	*FOXP1*	71429509	71572956	143447	3p13
RP11-69L8	*FOXP1*	71572153	71733818	161665	3p13
RP11-113M16	*RPPH1*	20794573	20979309	184736	14q11.2
RP11-147O12	*LRBA*	151439748	151578761	139013	4q31.3
RP11-156N13	*LRBA*	151158076	151334332	176256	4q31.3
RP11-833H23	*LRBA*	151273194	151442964	169770	4q31.3
RP11-265A4	*LRBA*	151499319	151674156	174837	4q31.3
RP11-28E24	*LRBA*	151627684	151771649	143965	4q31.3
RP11-465P24	*LRBA*	151736644	151899786	163142	4q31.3
RP11-125M5	*LRBA*	151832217	152014630	182413	4q31.3
RP11-591L23	*SLC39A6*	33683777	33866984	183207	18q12.2
RP11-592J1	*ANKRD30A*	37294438	37461431	166993	10p11.21
RP11-264O24	*ANKRD30A*	37294438	37461431	166993	10p11.21
RP11-81D7	*DDX5*	62460761	62636156	175395	17q23.3 - 17q24.1
RP11-661B17	*CLTC*	57651039	57855337	204298	17q23.1
RP11-655A7	*COL3A1*	189708272	189880147	171875	2q32.2
RP11-639O1	*ETV6*	11737815	11916241	178426	12p13.2
RP11-418C2	*ETV6*	11916329	12086277	169948	12p13.2
RP11-91O9	*WWOX*	78053568	78205164	151596	16q23.1
RP11-72G1	*WWOX*	78147069	78342268	195199	16q23.1
RP11-345K17	*WWOX*	78275875	78520559	244684	16q23.1
RP11-264L1	*WWOX*	78487649	78638009	150360	16q23.1
RP11-994F22	*WWOX*	78600645	78793477	192832	16q23.1
RP11-1062G14	*WWOX*	78837285	79023146	185861	16q23.1
RP11-298E11	*WWOX*	79018177	79221790	203613	16q23.1 - 16q23.2
RP11-101D10	*WWOX*	79194633	79393394	198761	16q23.1 - 16q23.2

Supplementary Table 4: Putative driver fusion events in 8p11-p12 amplified breast carcinomas

Sample	Fusion	Fusion breakpoints	5′ gene in CDS?	5′ segment type	3′ gene in CDS?	3′ segment type	Adjusted *p*-value	Driver Bayesian probability
10068	*ABL2-IGFBP5*	chr1:179073904>chr2:217539667	No	Intron	No	Intron	4.02E-04	0.999990417
10400	*ADAR-PI15*	chr1:154560641>chr8:75765846	Yes	Exon	No	Intron	0.001203145	0.999978125
8931	*ASB7-MEF2A*	chr15:101152632>chr15:100138635	Yes	Exon	No	Intron	0.003786331	0.999708744
8931	*ASB7-MEF2A*	chr15:101152632>chr15:100173183	Yes	Exon	No	Intron	0.003786331	0.999708744
7325	*ATAD2-INSR*	chr8:124361525>chr19:7152938	Yes	Exon	Yes	Exon	0.018215303	0.998344063
10408	*B2M-ETV6*	chr15:45010150>chr12:11988371	No	Intron	Yes	Intron	2.90E-05	0.999995858
11315	*BCL2-IGFBP5*	chr18:60983420>chr2:217540036	Yes	Intron	No	Intron	0.040741279	0.978874892
11315	*BCL2-IGFBP5*	chr18:60983421>chr2:217540037	Yes	Intron	No	Intron	0.040741279	0.978874892
11315	*BCL2-RHOBTB3*	chr18:60981376>chr5:95130935	Yes	Intron	No	Intron	0.040741279	0.978874892
10068	*CLTC-COL3A1*	chr17:57760118>chr2:189877010	Yes	Exon	No	Intron	0.215073028	0.964154495
10569	*COL12A1-WWP1*	chr6:75822948>chr8:87423956	Yes	Exon	Yes	Exon	0.429016568	0.877423838
10569	*COL12A1-WWP1*	chr6:75822948>chr8:87423957	Yes	Exon	Yes	Exon	0.429016568	0.877423838
10068	*COL1A2-MACF1*	chr7:94057752>chr1:39856521	Yes	Exon	Yes	Intron	0.36978708	0.929564366
10068	*COL1A2-TRPS1*	chr7:94059813>chr8:116421597	No	Intron	No	Intron	0.198345903	0.981109914
10569	*COL1A2-TRPS1*	chr7:94059788>chr8:116662316	No	Intron	No	Intron	7.78E-04	0.999972202
10400	*CPB1-TRPS1*	chr3:148558738>chr8:116426569	Yes	Exon	Yes	Exon	0.124155579	0.986455755
11659	*DDX17-TRPS1*	chr22:38888654>chr8:116681014	Yes	Intron	No	Intron	5.85E-04	0.999883024
11659	*DDX17-TRPS1*	chr22:38888655>chr8:116681015	Yes	Intron	No	Intron	5.85E-04	0.999883024
11315	*DDX5-WWOX*	chr17:62496139>chr16:78589835	Yes	Exon	Yes	Intron	0.012819635	0.996676391
11315	*DDX5-WWOX*	chr17:62496141>chr16:78589833	Yes	Exon	Yes	Intron	0.012819635	0.996676391
10068	*DDX6-COL1A2*	chr11:118619225>chr7:94060368	No	Intron	No	Intron	0.199591359	0.976239124
10068	*DDX6-COL1A2*	chr11:118619227>chr7:94060366	No	Intron	No	Intron	0.199591359	0.976239124
11315	*DEPTOR-ZNF704*	chr8:120977650>chr8:81733850	Yes	Exon	No	Intron	0.01438201	0.995738664
11378	*ERBB2-AHNAK*	chr17:37882045>chr11:62288082	Yes	Exon	Yes	Exon	0.1799945	0.955001375
11378	*ERBB2-AHNAK*	chr17:37882044>chr11:62288083	Yes	Exon	Yes	Exon	0.1799945	0.955001375
11378	*ERBB2-CTNNA1*	chr17:37868282>chr5:138260947	Yes	Exon	Yes	Exon	0.1799945	0.955001375
11378	*ERBB2-LUC7L3*	chr17:37882358>chr17:48829549	Yes	Intron	No	Intron	0.164758186	0.991762091
8931	*ESR1-C6orf211*	chr6:152201906>chr6:151775683	Yes	Exon	Yes	Exon	0.002959268	0.999317092
8931	*ESR1-C6orf211*	chr6:152201910>chr6:151775683	Yes	Intron	Yes	Exon	0.002959268	0.999317092
11315	*ESR1-IGFBP5*	chr6:152419999>chr2:217537385	Yes	Exon	No	Intron	0.003023309	0.999888026
11315	*ESR1-IGFBP5*	chr6:152201886>chr2:217539806	Yes	Exon	No	Intron	0.111414206	0.929850315
10400	*FGFR1-CPB1*	chr8:38270916>chr3:148562497	No	Intron	Yes	Exon	0.002699831	0.999901824
10400	*FGFR1-PI15*	chr8:38277867>chr8:75757445	Yes	Intron	Yes	Exon	0.021172465	0.998845138
10569	*FN1-TRPS1*	chr2:216243914>chr8:116426876	Yes	Exon	Yes	Exon	9.65E-04	0.999931045
10569	*FN1-TRPS1*	chr2:216274787>chr8:116538622	Yes	Exon	Yes	Intron	0.019775538	0.995056115
10569	*FN1-TRPS1*	chr2:216274788>chr8:116538623	Yes	Exon	Yes	Intron	0.019775538	0.995056115
10068	*IGFBP5-ABL2*	chr2:217537840>chr1:179076778	No	Intron	No	Intron	0.089924551	0.995717879
11315	*IGFBP5-CA12*	chr2:217539466>chr15:63616867	No	Intron	No	Intron	0.011561728	0.995717879
11315	*IGFBP5-CA12*	chr2:217538433>chr15:63616872	No	Intron	No	Intron	0.011561728	0.995717879
11315	*IGFBP5-ESR1*	chr2:217537729>chr6:152421653	No	Intron	No	Intron	0.002774398	0.999486223
11315	*IGFBP5-MACF1*	chr2:217542904>chr1:39753105	Yes	Exon	Yes	Exon	0.017717691	0.992781681
11315	*IGFBP5-TMEM64*	chr2:217541084>chr8:91636460	No	Intron	No	Intron	0.002774398	0.999486223
8629	*LYRM4-SFMBT2*	chr6:5216851>chr10:7327916	Yes	Exon	Yes	Exon	0.467271142	0.875394362
10408	*MACF1-YWHAZ*	chr1:39952004>chr8:101932726	No	Intron	No	Intron	0.241265449	0.931067015
11372	*MAST2-PRKCA*	chr1:46425149>chr17:64683229	Yes	Exon	Yes	Exon	0.139249908	0.988395841
11378	*MAST2-PRKCA*	chr1:46425149>chr17:64683229	Yes	Exon	Yes	Exon	0.11604159	0.988395841
11659	*MGP-TRPS1*	chr12:15038695>chr8:116426080	Yes	Exon	No	Intron	0.023167852	0.988416074
11659	*MGP-TRPS1*	chr12:15035072>chr8:116616544	Yes	Exon	Yes	Exon	0.023167852	0.988416074
11315	*MMP16-ZNF34*	chr8:89053096>chr8:146003910	No	Intron	Yes	Exon	0.470695754	0.668769654
11315	*MMP16-ZNF34*	chr8:89058897>chr8:146003910	Yes	Exon	Yes	Exon	0.470695754	0.668769654
11315	*MMP16-ZNF34*	chr8:89053965>chr8:146003910	Yes	Exon	Yes	Exon	0.470695754	0.668769654
10823	*MTF1-SLCO1B1*	chr1:38304297>chr12:21325584	Yes	Exon	Yes	Exon	0.027132269	0.997193214
10823	*MTF1-SLCO1B1*	chr1:38304297>chr12:21329710	Yes	Exon	Yes	Exon	0.027132269	0.997193214

10823	*MTUS1-TRPS1*	chr8:17601113>chr8:116430680	Yes	Exon	Yes	Exon	0.00281909	0.99990279
10569	*NBN-COL3A1*	chr8:90971025>chr2:189839203	Yes	Exon	No	Intron	0.00283485	0.999696266
7253	*NCK1-CLSTN2*	chr3:136647069>chr3:139894793	Yes	Exon	Yes	Exon	0.00958784	0.99520608
10569	*NFKBIZ-FN1*	chr3:101574901>chr2:216260274	Yes	Intron	Yes	Intron	0.443035777	0.841772937
10823	*NUDCD1-RAD54B*	chr8:110283234>chr8:95479783	Yes	Exon	No	Intron	0.124243172	0.978578764
10400	*PI15-TRPS1*	chr8:75763042>chr8:116617015	No	Intron	Yes	Exon	0.034412409	0.997497279
10068	*PLXDC2-FN1*	chr10:20569819>chr2:216279615	No	Intron	Yes	Exon	0.170504595	0.9878211
11315	*PSPC1-TRPS1*	chr13:20304379>chr8:116430680	Yes	Exon	Yes	Exon	0.005592611	0.999585733
8629	*RAB2A-CHD7*	chr8:61504528>chr8:61653818	Yes	Exon	No	Intron	0.007969021	0.998937464
8629	*RAB2A-CHD7*	chr8:61484672>chr8:61693559	Yes	Exon	Yes	Exon	0.007969021	0.998937464
8931	*RIN2-SLC24A3*	chr20:19972943>chr20:19496132	Yes	Exon	Yes	Exon	0.227555444	0.912478675
8629	*RPAIN-RABEP1*	chr17:5331531>chr17:5250085	Yes	Exon	Yes	Exon	0.416198058	0.861267314
10415	*SKIL-PRRC2B*	chr3:170110915>chr9:134330495	No	Intron	Yes	Exon	0.434368897	0.945703888
11659	*SLC39A8-TRPS1*	chr4:103183528>chr8:116631981	No	Intron	Yes	Exon	0.001428648	0.999571406
11659	*SLC39A8-TRPS1*	chr4:103183527>chr8:116631980	No	Intron	Yes	Exon	0.001428648	0.999571406
10569	*SPARC-MGP*	chr5:151042777>chr12:15035003	No	Intron	No	Intron	0.004188382	0.999252075
11315	*TMEM64-ZNF704*	chr8:91637222>chr8:81672849	No	Intron	Yes	Intron	0.003857349	0.999571406
11315	*TRMT1L-RALGPS2*	chr1:185113038>chr1:178745817	Yes	Exon	No	Intron	0.017114966	0.992393349
8860	*TRPS1-ANKRD30A*	chr8:116425696>chr10:37430974	No	Intron	Yes	Exon	0.145727334	0.995776019
11659	*TRPS1-C6orf211*	chr8:116599677>chr6:151790689	Yes	Exon	No	Intron	0.141932844	0.886453724
10400	*TRPS1-CPB1*	chr8:116616434>chr3:148567056	Yes	Exon	Yes	Intron	0.093772173	0.991475257
10569	*TRPS1-FN1*	chr8:116595188>chr2:216256401	Yes	Intron	Yes	Exon	0.00344624	0.99950768
10823	*TRPS1-HECTD1*	chr8:116465658>chr14:31576001	Yes	Intron	Yes	Exon	0.470405999	0.886453724
8860	*TRPS1-IQGAP1*	chr8:116427260>chr15:91029819	Yes	Exon	Yes	Intron	0.472858604	0.97944093
10823	*TRPS1-MTUS1*	chr8:116617104>chr8:17651601	Yes	Exon	No	Intron	0.020401604	0.998592993
8860	*TRPS1-NF1*	chr8:116599672>chr17:29435023	Yes	Exon	Yes	Intron	0.020096678	0.999708744
10400	*TRPS1-RNF213*	chr8:116616836>chr17:78263532	Yes	Exon	Yes	Exon	0.161535548	0.97944093
11659	*WARS2-DDX4*	chr1:119683178>chr5:55041992	Yes	Exon	Yes	Exon	0.132521351	0.907235054
11315	*WWOX-DDX5*	chr16:78910763>chr17:62500878	Yes	Intron	Yes	Exon	0.023494565	0.988687802
8629	*ZFAND3-ATRX*	chr6:37897775>chrX:76972720	Yes	Exon	Yes	Exon	0.006428915	0.999571406

The adjusted *p*-value is the Bayesian probability of the fusion being a passenger event and the Bayesian driver probability is the probability of the fusion being a driver event. Fusion events with adjusted $P < 0.5$ were classified as “driver” fusions.
